# Isolation, Characterization, Crystal Structure Elucidation of Two Flavanones and Simultaneous RP-HPLC Determination of Five Major Compounds from *Syzygium campanulatum* Korth

**DOI:** 10.3390/molecules200814212

**Published:** 2015-08-04

**Authors:** Abdul Hakeem Memon, Zhari Ismail, Fouad Saleih Resq Al-Suede, Abdalrahim F. A. Aisha, Mohammad Shahrul Ridzuan Hamil, Mohammed Ali Ahmed Saeed, Madeeha Laghari, Amin Malik Shah Abdul Majid

**Affiliations:** 1 Department of Pharmaceutical Chemistry, School of Pharmaceutical Sciences, Universiti Sains Malaysia, 11800-Minden, Penang, Malaysia; E-Mails: memon_h79@yahoo.com (A.H.M.); abedaisheh@yahoo.com (A.F.A.A.); shahrul.ridzuan18@gmail.com (M.S.R.H.); mohali141@yahoo.com (M.A.A.S.); 2 EMAN Research and Testing Laboratory, School of Pharmaceutical Sciences, Universiti Sains Malaysia, 11800-Minden, Penang, Malaysia; E-Mails: fsr2009@yahoo.com (F.S.R.A.-S.); aminmalikshah@gmail.com (A.M.S.A.M.); 3 Department of Pharmaceutical Technology, School of Pharmaceutical Sciences, Universiti Sains Malaysia, 11800-Minden, Penang, Malaysia; E-Mail: fairy_rose25@yahoo.com

**Keywords:** *Syzygium campanulatum* Korth, secondary metabolites, X-ray crystallography, RP-HPLC

## Abstract

Two flavanones named (2*S*)-7-Hydroxy-5-methoxy-6,8-dimethyl flavanone (**1**), (*S*)-5,7-dihydroxy-6,8-dimethyl-flavanone (**2**), along with known chalcone, namely, (*E*)-2ʹ,4ʹ-dihydroxy-6ʹ-methoxy-3ʹ,5ʹ-dimethylchalcone (**3**) and two triterpenoids, namely, betulinic and ursolic acids (**4** and **5**), were isolated from the leaves of *Syzygium campanulatum* Korth (Myrtaceae). The structures of compounds (**1** and **2**) were determined on the basis of UV-visible, FTIR, NMR spectroscopies and LC-EIMS analytical techniques. Furthermore, new, simple, precise, selective, accurate, highly sensitive, efficient and reproducible RP-HPLC method was developed and validated for the quantitative analysis of the compounds (**1**–**5**) from *S. campanulatum* plants of five different age*.* RP-HPLC method was validated in terms of specificity, linearity (*r*^2^ ≤ 0.999), precision (2.0% RSD), and recoveries (94.4%–105%). The LOD and LOQ of these compounds ranged from 0.13–0.38 and 0.10–2.23 µg·mL^−1^, respectively. Anti-proliferative activity of isolated flavanones (**1** and **2**) and standardized extract of *S. campanulatum* was evaluated on human colon cancer (HCT 116) cell line. Compounds (**1** and **2**) and extract revealed potent and dose-dependent activity with IC_50_ 67.6, 132.9 and 93.4 µg·mL^−1^, respectively. To the best of our knowledge, this is the first study on isolation, characterization, X-ray crystallographic analysis of compounds (**1** and **2**) and simultaneous RP-HPLC determination of five major compounds (**1**–**5**) from different age of *S. campanulatum* plants.

## 1. Introduction

The *Syzygium* genus (Myrtaceae) comprises about 1200 species, and has high levels of diversity. It is widely distributed from Malaysia to north-eastern Australia [1]. It is reported that the genus *Syzygium* is rich in secondary metabolites such as terpenoids [2,3], phenylpropanoids [4], chalcones [5], flavonoids [6], lignans [7], alkyl phloroglucinols [8], hydrolysable tannins and chromone derivatives [9]. Therapeutically, *Syzygium* genus is used in the treatment of rheumatism [10], haemorrhage, GIT disorders [11], diabetes [12], inflammation, allergy [13,14], convulsion [15], hypertension [16] and bacterial infections [17]. Despite its wide distribution, scientific research about *S. campanulatum* is scarce. Memon *et al.*, 2014 reported isolation, characterization, crystal structure elucidation, and anticancer effect of dimethyl cardamonin isolated from *Syzygium campanulatum* Korth [5]. Aisha *et al.*, 2013 reported anti-angiogenesis and antitumor effects of *Syzygium campanulatum* extracts [18]. Furthermore, previous work conducted by our research group indicated *Syzygium aromaticum* extracts as a good source of betulinic acid with potential anti-breast cancer effect [3]. The present study was conducted in order to further analyze the phytochemical profile of *S. campanulatum* including: isolation and characterization of new compounds from leaves extracts and development of reverse phase high performance liquid chromatography (RP-HPLC) method for the quantitative determination of the major secondary metabolites in *S. campanulatum* extracts. It was reported that the environmental conditions affect the formation of secondary metabolites which are found mostly in young and actively growing tissues [19]. Therefore, this study also aimed to investigate the effect of age of *S. campanulatum* shrub on the concentration of its active ingredients. The isolated compounds were characterized by HPLC, LCMS, X-ray crystallography and NMR. A new, rapid, accurate, precise, robust and reproducible RP-HPLC method has been developed and validated for simultaneous determination of five major compounds in *S. campanulatum* extracts. The developed HPLC method was applied in studying the effect of plant’s age on the concentration of its major compounds.

## 2. Results and Discussion

The crude extract *n*-hexane:methanol (1:1) of dried green leaves of *S. campanulatum* was extracted using soxhletion. The crude extract was subjected to flash column chromatography using increasing concentration of ethyl acetate in *n*-hexane starting with 100% *n*-hexane. Nineteen fractions were obtained: the white, yellow, yellow orange and white solid appeared in fractions 16–17, 6, 7–8 and 12–14 (obtained in *n*-hexane:ethyl acetate 60:40, 92:8, 85:15, and 60:40), respectively. All solid compounds were re-crystallized using a mixture of acetone, methanol: *n*-hexane and methanol. White, yellow, yellow orange and white needle type crystals of compounds (**1**–**4**), respectively were obtained.

**Figure 1:**
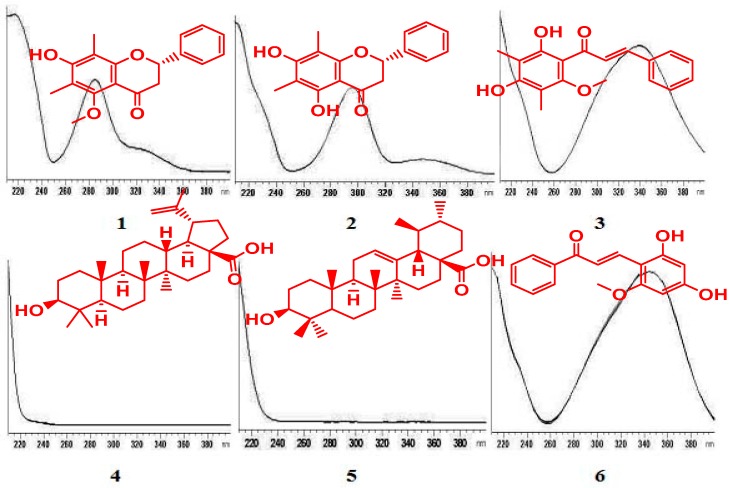
UV-spectra of compounds: (2*S*)-7-Hydroxy-5-methoxy-6,8-dimethyl flavanone (**1**), (*S*)-5,7-dihydroxy-6,8-dimethyl flavanone (**2**), (*E*)-2ʹ,4ʹ-dihydroxy-6ʹ-methoxy-3ʹ,5ʹ-dimethylchalcone (**3**), Betulinic acid (**4**) Ursolic acid (**5**) and (*E*)-2ʹ,4ʹ-dihydroxy-6ʹ-methoxy chalcone (**6**).

The UV-Vis spectra of compounds (**1**–**5**) showed absorption at λ_max_ 330 and 210 nm (Figure 1).

**Figure 2:**
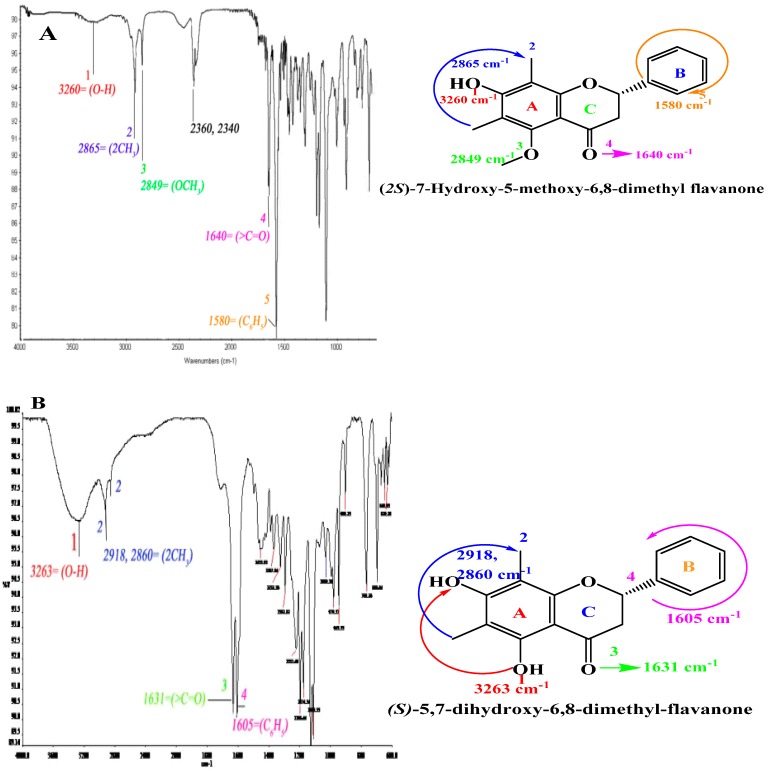
FTIR-spectra of compounds: (2*S*)-7-Hydroxy-5-methoxy-6,8-dimethyl flavanone (**A**) and (*S*)-5,7-dihydroxy-6,8-dimethyl flavanone (**B**).

FTIR spectrum of compound (**1** and **2**) showed a strong and sharp vibrational band at 3260–3263 cm^−1^ that indicated the presence of (O-H stretching) [20], 1640–1631 cm^−1^ (C=O), 1580–1605 cm^−1^ (C_6_H_5_). The presence of alkyl groups was assigned by 2860, 2918 cm^−1^ two vibrational bands in compound (**1**) and 2865 cm^−1^ in compound (**2**) [21]. A doublet was found at 2360 and 2340 cm^−1^ in compound (**1**) may be due to presence of small amount of CO_2_ in sample chamber when data was collected [22]. Furthermore, the presence of methoxy group was assigned by 2849 cm^−1^ in compound (**1**) [23]. These prominent characteristic functional groups indicated the presence of flavanone, based upon a 15-carbon skeleton consisting of two benzene rings (A and B) linked via a heterocyclic pyran ring (C) [24] Figure 2.

**Figure 3:**
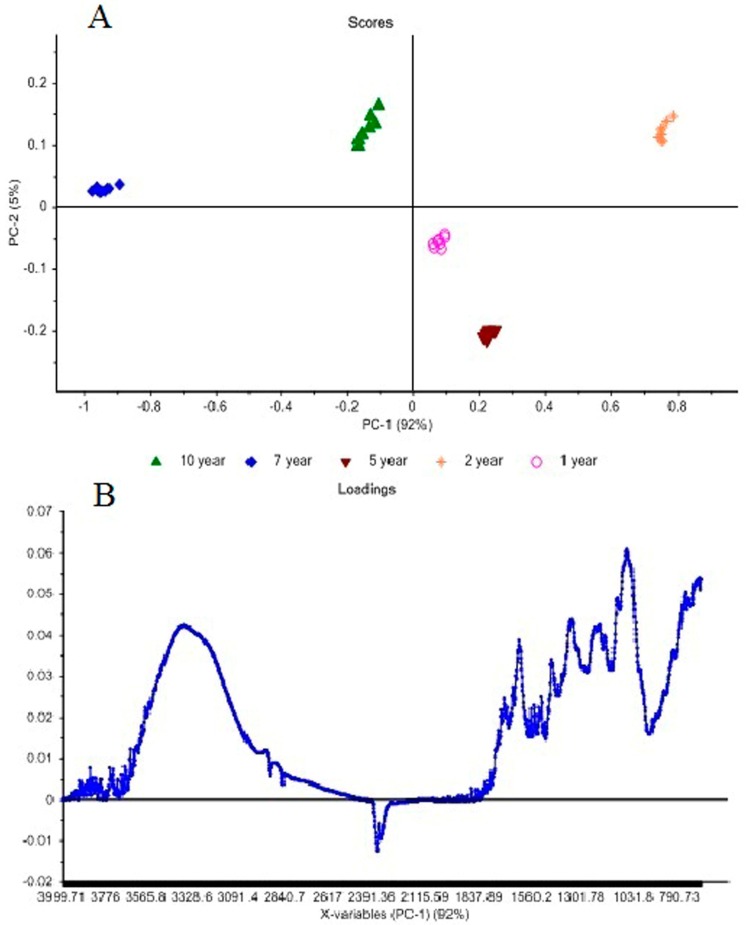
Principle Component Analysis (PCA) Score Plot (**A**) and Loading Plot (**B**) of *S. campanulatum* plants of different age.

Principle component analysis (PCA) of five different age plants (**A**–**C**) has shown that all extracts have different region of absorption because of the different concentration of compounds (**1**–**5**) Figure 3. PCA data can be used to differentiate age of plant with maximum concentration of all compounds (**1**–**5**).

**Figure 4:**
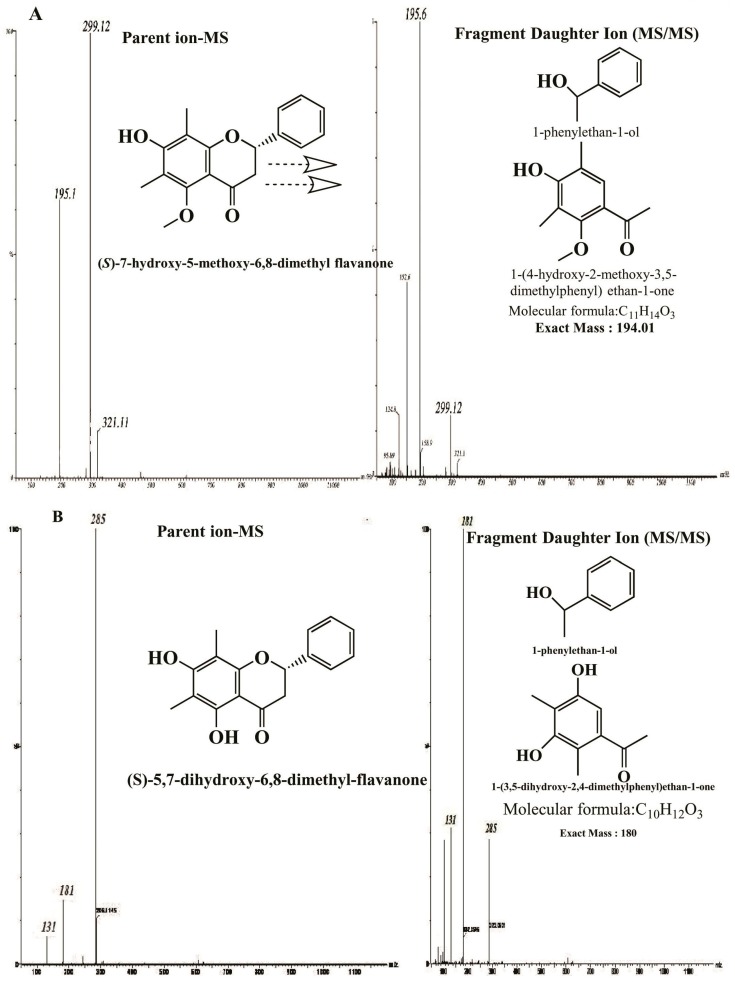
LC-EIMS-spectra of compounds: (2*S*)-7-Hydroxy-5-methoxy-6,8-dimethyl flavanone (**A**) and (*S*)-5,7-dihydroxy-6,8-dimethyl flavanone (**B**).

LC-EIMS analysis of compound (**1** and **2**) indicated molecular formulae C_18_H_18_O_4_ (*m*/*z* 298.10 calc. [M]^+^ 299.10), and C_17_H_16_O_4_ (*m*/*z* 284.10 calc. [M]^+^ 285.10), respectively. The molecular weights of compounds (**1** and **2**) were determined by liquid chromatography-mass spectroscopy (LC-MS). Compound (**2**) showed proposed retrocyclization cleavage of molecule [25]. The fragmented molecular ion peaks were observed at 195 and 181 as daughter ions and the main fragmentation peaks at 299 and 285 for compounds (**1** and **2**), respectively (Figure 4).

Compounds (**1** and **2**) were also characterized by ^13^C, ^1^H, ^13^C-DEPTQ-135, -145 and -90, 2D-HSQC, 2D-HMBC2D-TOCSY and 2D-NOESY NMR spectra (supplementary data, Figures S1–S12). ^1^H-NMR spectrum of compound (**1**) showed signals at δ: 5.3 (1H, dd, *J* = 9.9 and 15.65 Hz, H-2), 2.6 & 2.8 (2H, dd, *J* = 3.00 and 16.7 Hz, H-3), 7.4 (1H, d, *J* = 7.5 Hz, H-2ʹ), 7.3 (1H, t, *J* = 7.6 Hz, H-3′), 7.2 (1H, t, *J* = 7.0 Hz, H-4′), 12.1 (1H, s, H-O), 12.0 (1H, s, H-O), 3.6 (3H, s, H-OMe), 1.98 (3H, s, H-Me) and 2.0 (3H, s, H-Me), compound (**2**): at δ: 5.3 (1H, dd, *J* = 9.5 and 15.7 Hz, H-2), 2.9 & 2.7 (2H, dd, *J* = 3.18 and 17.0 Hz, H-3), 7.4 (1H, d, *J* = 7.2 Hz, H-2′), 7.3 (1H, t, *J* = 7.4 Hz, H-3′), 7.2 (1H, t, *J* = 9.5 Hz, H-4′), 12.1 (1H, s, H-O) 12.0 (1H, s, H-O), 1.9 (3H, s, H-Me) and 1.91 (3H, s, H-Me), respectively (supplementary data, Figures S1 and S7).

**Figure 5:**
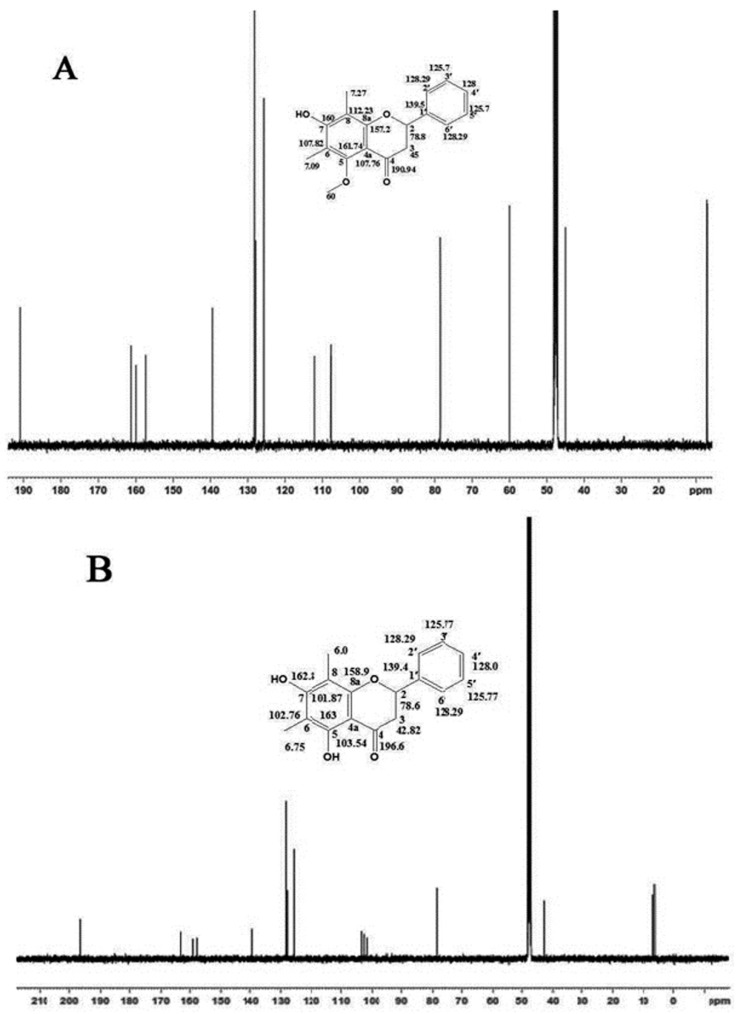
^13^C-NMR-spectra of compounds: (2*S*)-7-Hydroxy-5-methoxy-6,8-dimethyl flavanone (**A**) and (*S*)-5,7-dihydroxy-6,8-dimethyl flavanone (**B**).

^13^C-NMR spectrum: ^13^C-NMR spectrum of compound (**1**) showed signals at δ: C-2 (78.8), C-3 (45.0), C-4 (190.94), C-4a (107.76), C-5 (161.74), C-5-O-CH_3_ (60.0), C-6 (107.82), C-7 (160.0), C-8 (112.23), C-8a (157.2), C-1′ (139.5), C-2′ & C6′ (128.29), C-3′ & C-5′ (125.7), C-4′ (128), C-6-CH_3_ (7.09) and C-8-CH_3_ (7.27) Figure 5A.

^13^C-NMR spectrum: ^13^C-NMR spectrum of compound (**2**) showed signals at δ: C-2 (78.6), C-3 (42.82), C-4 (196.6), C-4a (103.54), C-5 (163.0), C-6 (102.76), C-7 (162.8), C-8 (101.87), C-8a (158.9), C-1′ (139.4), C-2′ & C6′ (128.29), C-3′ & C-5′ (125.77), C-4′ (128.0), C-6-CH_3_ (6.75) and C-8-CH_3_ (6.0) Figure 5B.

It was observed that the ^13^C-DEPT-135 shows positive CH_3_ and CH_2_ and negative CH_2_ signals and vice versa in -90 NMR spectra recorded in MeOD at 125.75 MHz at 25 °C for both compounds (**1** and **2**). 2D-HSQC and HMBC-NMR spectra of compounds (**1** and **2**) demonstrate that C-2 has correlation with C-3, C-4 and C-1′. 2D-TOCSY and 2D-COSY NMR spectra illustrate the characteristic diagonal component and cross peaks of compounds (**1** and **2**) found in respective spectra (Refer to the supplementary data, Figures S2–S12).

**Figure 6:**
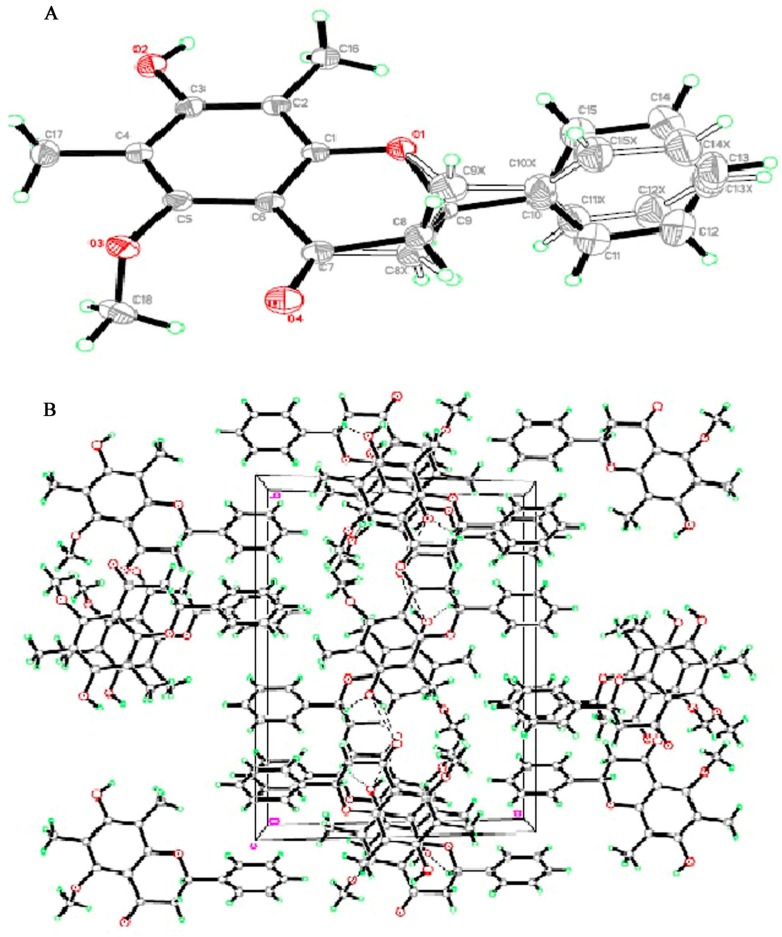
(**A**) Stereochemical structures of (2*S*)-7-Hydroxy-5-methoxy-6,8-dimethyl flavanone; (**B**) Crystal packing of (2*S*)-7-Hydroxy-5-methoxy-6,8-dimethyl flavanone. The molecules packed in monoclinic crystal system through intermolecular hydrogen bonds shown as dashed lines.

**Figure 7:**
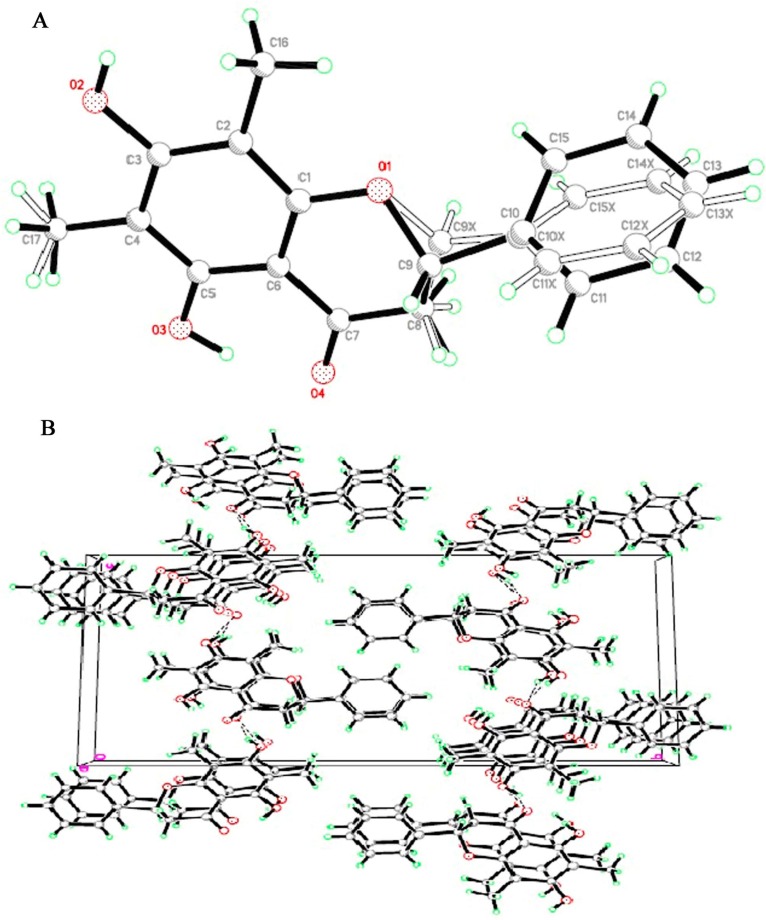
(**A**) Stereochemical structures of (*S*)-5,7-dihydroxy-6,8-dimethyl flavanone; (**B**) Crystal packing of (*S*)-5,7-dihydroxy-6,8-dimethyl flavanone. The molecules packed in monoclinic crystal system through intermolecular hydrogen bonds shown as dashed lines.

Separate single crystals of compounds (**1** and **2**) were found appropriate for X-ray crystallographic study. The crystals of compounds (**1** and **2**) appeared as white and yellow needle type structures, respectively. A perspective view of the crystal structures of compounds (**1** and **2**) are shown in Figure 6A and Figure 7A. The intra-molecular geometry aspects of both compounds (**1** and **2**) were analyzed using Monoclinic space group P21 (No. 4) a knowledge base molecular geometry obtained from Cambridge Structural Database. Each unit of compound (**1** and **2**) consists of two benzene rings (A and B) linked by a heterocyclic pyran ring (C). In addition one hydroxyl, methoxy and two methyl groups in compound (**1**), two hydroxyl and methyl groups in compound (**2**) are attached on one benzene ring. Crystal data and structure refinement details of compound (**1** and **2**) is; Formulae: C_18_H_18_O_4_ and C_17_H_16_O_4_, molecular weight: 298 and 284, crystal system: monoclinic, space group: P21 (No. 4).The crystals refinement data of compounds (**1** and **2**) are shown in Table 1, whereas the selected bond lengths and angles of both compounds (**1** and **2**) crystallographically are shown in Figure 6 and Figure 7 and are also listed in (Supplementary data, Tables S1–S18).

**Table 1:** Crystal data and structure refinement details for (2*S*)-7-hydroxy-5-methoxy-6,8-dimethyl flavanone and (*S*)-6,8-dihydroxy-5,7-dimethyl flavanone.

Parameters	Crystal Data	
	Compound 1	Compound 2
Formula	C_18_H_18_O_4_	C_17_H_16_O_4_
Formula Weight	298.32	284.30
Crystal System	Monoclinic	Monoclinic
Space group	P21/c (No. 14)	P21/c (No. 14)
Alpha (°)	90	90
Beta (°)	105.966 (3)	100.1616 (17)
Gamma (°)	90	90
Unit cell dimensions a (A°)	12.7683 (14)	4.8133 (1)
b (A°)	17.2730 (15)	24.5685 (6)
c (A°)	7.2728 (7)	12.7303 (4)
V (A°3)	1542.1 (3)	1481.82 (7)
Z	4	4
Density (calcd) (gm/cm^3^)	1.285	1.274
Mu(CuKa) [/mm]	0.090	0.744
F(000)	632	600
Crystal Size (mm)	0.479 × 0.167 × 0.122	0.03 × 0.09 × 0.32
Temperature (K)	294	294
Radiation (A°)	MoKa	CuKa 1.54178
θ Min, max (°)	1.659, 27.499	4.0, 70.2
Dataset	−16:16; −22:22; −8: 9	−5:5; −28:29; −15:14
Tot., Uniq. Data	15,650, 3514	26,315, 2751
R (int)	0.0416	0.037
Observed data [I > 2.0 sigma(I)]	2558	2104
Nref, Npar	3514, 240	2751, 240
R, wR2, S	0.0775, 0.2521, 0.975	0.056, 0.1714, 1.10

S = Goodness of fit, in general value of S should be close to 1. wR2 = Weighted Residual Factor is the most closely related to the refinement against squared structure factors. The weighting factor w is individually derived from the standard uncertainties of the measured reflections and expresses the confidence we have in every single reflection. This factor should be minimum during refinement. R = Un-weighted residual factor. This factor should also be minimum during refinement. Nref = Number of independent reflections. Npar = the number of refined parameters. R (int) = merging residual value, reflects the summations involve all the input reflections for which more than one symmetry equivalent are averaged.

The crystal packing of both compounds (**1** and **2**) shows that the compounds (**1** and **2**) molecules are connected via intermolecular hydrogen bonding interactions in which C7 of aromatic ring (A) as donor for H-bond via C4-O of pyran ring (C) in the neighboring molecules at –x + 1, −y, −z + 1 to produce a three-dimensional network (Figure 6B and Figure 7B). The compounds (**1** and **2**) showed statistical conformational disorders, with three conformations having two orientations of alkyl group attached on C-6 of aromatic ring (A) of compound (**1**) and three orientations of the phenyl ring (B) and two orientations of the fused heterocyclic pyran ring (C) of compounds (**1** and **2**). The crystal stability of compounds (**1** and **2**) is assumed to be improved by weak intermolecular interactions. Furthermore, π-π interactions are present between the aromatic rings of neighboring chroman-4-one groups with the same conformation at (−x + 1, −y, −z + 1). The isomerization equilibrium between the flavanone and chalcone forms can be correlated with this conformational disorder [26]. This conformational behavior has major impact on the diverse biological activities of this class of compounds [27]. Crystal data and structure refinement details of compounds (**1** and **2**) are shown in Table 1. Some selected bond angles of compounds (**1** and **2**) are shown in Table 2 and complete data is given in supplementary data (Tables S1–S18). Crystallography data of compound (**1**) is reported which was obtained from aqueous ethanolic extract of the leaves of *C. guianensis*, using dichloromethane extraction and a silica-gel chromatographic column. Single crystals were obtained by evaporation from a chloroform solution [26].

**Table 2:** Selected bond angles (°) of (2*S*)-7-Hydroxy-5-methoxy-6,8-dimethyl flavanone and (*S*)-6,8-dihydroxy-5,7-dimethyl flavanone.

Compound-1		Compound-2	
Atoms	Bond Angles (°)	Atoms	Bond Angles (°)
C1-O1-C9	115.76(16)	C9-O1-C1-C2	154.0(3)
C2-C3-C4	123.12(16)	C1-O1-C9-C8	52.9(4)
C3-C4-C17	120.72(17)	C1-O1-C9-C10	172.7(3)
C4-C5-C6	122.19(16)	O1-C1-C2-C16	1.5(3)
C5-C4-C3	117.90(16)	C6-C1-C2-C3	2.1(3)
C1-C6-C5	116.81(15)	C1-C2-C3-O2	179.14(18)
C3-C2-C16	122.27(15)	C16-C2-C3-C4	178.51(19)
O2-C3-C2	121.25(16)	O2-C3-C4-C5	178.59(18)
C3-C4-C17	120.72(17)	C2-C3-C4-C17	179.1(2)
C3-C4-C5	117.90(16)	C3-C4-C5-C6	2.6(3)
C17-C4-C5	121.38(17)	C17-C4-C5-O3	1.8(3)
O3-C5-C6	120.01(15)	O3-C5-C6-C1	178.83(18)
O2--H1, O2--C3	0.88(3), 1.359(2)	O2--H1, O2--O4	0.87(3),1.91(4)
O3--C5, O3--C18	1.376(2), 1.425(3)	O3--H1, O3--O4	0.92(3),1.73(3)
C16--H16A	0.9600	C16--H16A--O1	0.9600, 2.3400
C17--H17A	0.9600	C17--H17A--O3	0.9600, 2.3500

In the current study, however, compound (**1**) is reported, which was obtained from *n*-hexane methanolic extract of the leaves of *S. campanulatum*, using *n*-hexane:ethyl acetate extraction and a silica-gel chromatographic column. Single crystals were obtained by evaporation from a methanol solution. It was observed that it shows different bond angles due to use of different solvates. The incorporation of solvents resulted in changes of the crystal symmetry, intermolecular arrangements, stoichiometry and hydrogen bonding interactions [28].

**Figure 8:**
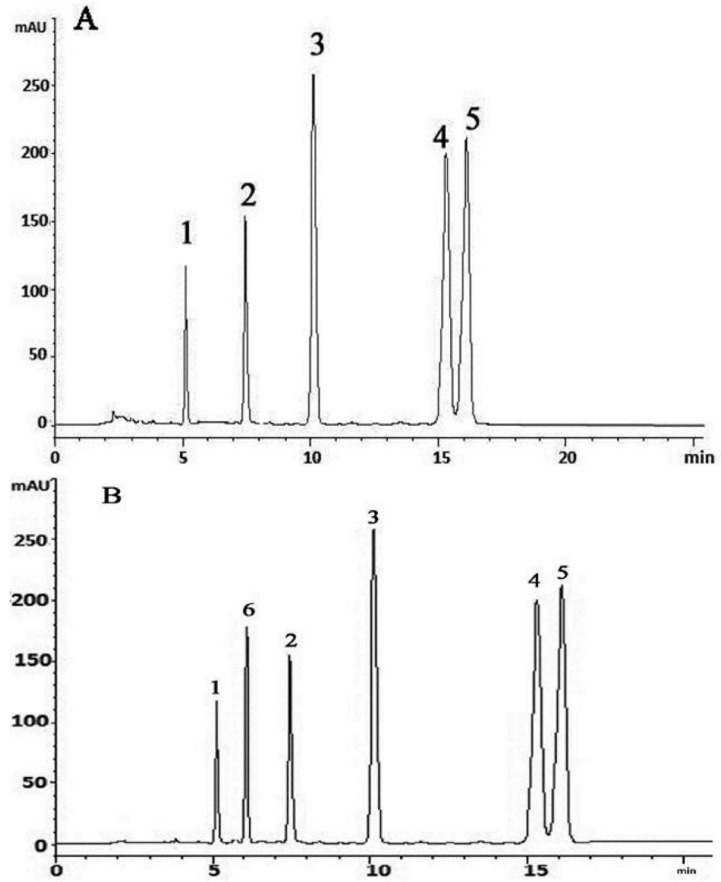
Representative HPLC chromatograms of; (**A**) of standards mixture; (**B**) With internal standards. (2*S*)-7-Hydroxy-5-methoxy-6,8-dimethyl flavanone (**1**), (*S*)-5,7-dihydroxy-6,8-dimethyl flavanone (**2**), (*E*)-2ʹ,4ʹ-dihydroxy-6ʹ-methoxy-3ʹ,5ʹ-dimethylchalcone (**3**), Betulinic acid (**4**) Ursolic acid (**5**) and (*E*)-2ʹ,4ʹ-dihydroxy-6ʹ-methoxy chalcone (**6**).

In the present study, HPLC method has been developed and validated for simultaneous determination of flavanones, chalcone and triterpenoids. Complete separation of the compounds (**1**–**5**) was accomplished in less than 20 min and the method was successfully applicable to perform simultaneous determination of all five compounds from standard solutions and different age plant extracts. Initially the mixture of compounds (**1**–**5**) was analyzed using a mobile phase consisting of 0.1% *o*-phosphoric acid:methanol (40:60 *v*/*v*) at flow rate 1 mL·min^−1^. Under these conditions, the separation and peak symmetries were not satisfactory, so the mobile phase was changed to 0.1% *o*-phosphoric acid:acetonitrile (40:60 *v*/*v*) with a flow rate of 1 mL·min^−1^ at 210 nm. It was observed that using these conditions, peaks were separated and resolved with good symmetry and sharpness. Therefore mobile phase containing 0.1% *o*-phosphoric acid:acetonitrile (40:60 *v*/*v*) was chosen for the present study. The retention times for compounds (**1**–**5**) were 5.1, 7.3, 10.0, 15.0 and 16.0 min, respectively (Figure 8A).

The concentration of each compound (**1**–**5**) in the extracts of different age plants was calculated from the experimental peak areas by interpolation to standard calibration curves (Table 3).

**Table 3:** Amount found of compounds (**1**–**5**) in different plants.

Plant	Compound 1 (%)	Compound 2 (%)	Compound 3 (%)	Compound 4 (%)	Compound 5 (%)	Total (%)
**A**	0.12	0.34	0.09	49.33	5.27	55.16
**B**	1.39	0.34	5.03	60.49	5.70	72.97
**C**	2.99	0.52	8.97	34.54	2.85	49.89
**D**	2.04	1.00	9.37	7.355	2.46	22.24
**E**	0.30	0.20	0.19	52.23	4.01	56.94

Plant **A** (one year old), Plant **B** (two years old), Plant **C** (five years old), Plant **D** (seven years old), Plant **E** (ten years old).

The calibration curves were linear over the concentration range of 0.78–200, 12.5–200 and 3.1–200 µg·mL^−1^ for compounds (**1**–**3**), (**4**) and (**5**), respectively. The regression equations representing the calibration curves and their correlation coefficients are summarized in Table 4. The % RSD in precision studies was found to be 0.14%–0.29% (Intra-day) and 0.59%–0.76% (Inter-day). The LOQ was found to be 0.7346 µg·mL^−1^ and the LOD was found to be 0.2423 µg·mL^−1^ (Table 4). The % RSD in accuracy studies (Table 4) and robustness was found to be less than 2%, indicating that the method is precise, accurate and robust (Supplementary data, Tables S19 and S20). Reproducibility of developed method was analyzed and performed by another person measuring the intra-day and inter-day precisions of method on different instrument in another laboratory. It was observed that the difference in terms of % RSD is not more than 2. There were no significant differences between % RSD values for intra-day and inter-day precision, which indicates the method is reproducible.

**Table 4:** Validation parameters for compounds (**1**–**5**).

Parameters	1	2	3	4	5
Retention time (min)	5.1	7.3	10.0	15.1	16.0
Linearity range (µg·mL^−1^)	0.78–200	0.78–200	0.78–200	12.5–200	3.1–200
Slope	47.90	54.57	54.66	1.208	5.182
Intercept	31.89	−73.75	−81.22	−1.25	−11.60
Correlation coefficient	0.999	0.999	0.999	0.999	0.999
LOD (µg·mL^−1^)	0.13	0.03	0.05	0.73	0.38
LOQ (µg·mL^−1^)	0.40	0.10	0.14	2.23	1.17
Accuracy (%)	95–92	99–91	101–98	96–92	94–92

The % RSD of robustness was not more than 1 and no significant changes were observed. The results are shown in supplementary data (Table S19). Accuracy of developed method was determined at three concentration levels of compounds (**1**–**5**) *i.e*., 250, 125 and 62.5 µg·mL^−1^ (*n* = 3), and the percentage recoveries were calculated and summarized in (Table 4).

It was observed that cardamonin used as internal standard did not interfere with the chromatograms of compounds (**1**–**5**). However, a sharp, well-shaped separated peak indicates the specificity of the developed method. The result for specificity is shown in Figure 8B. In this developed method, there was no interference from cardamonin structurally related to chalcone and flavanone. So this RP-HPLC method can be used in the quality control department of herbal drugs.

**Figure 9:**
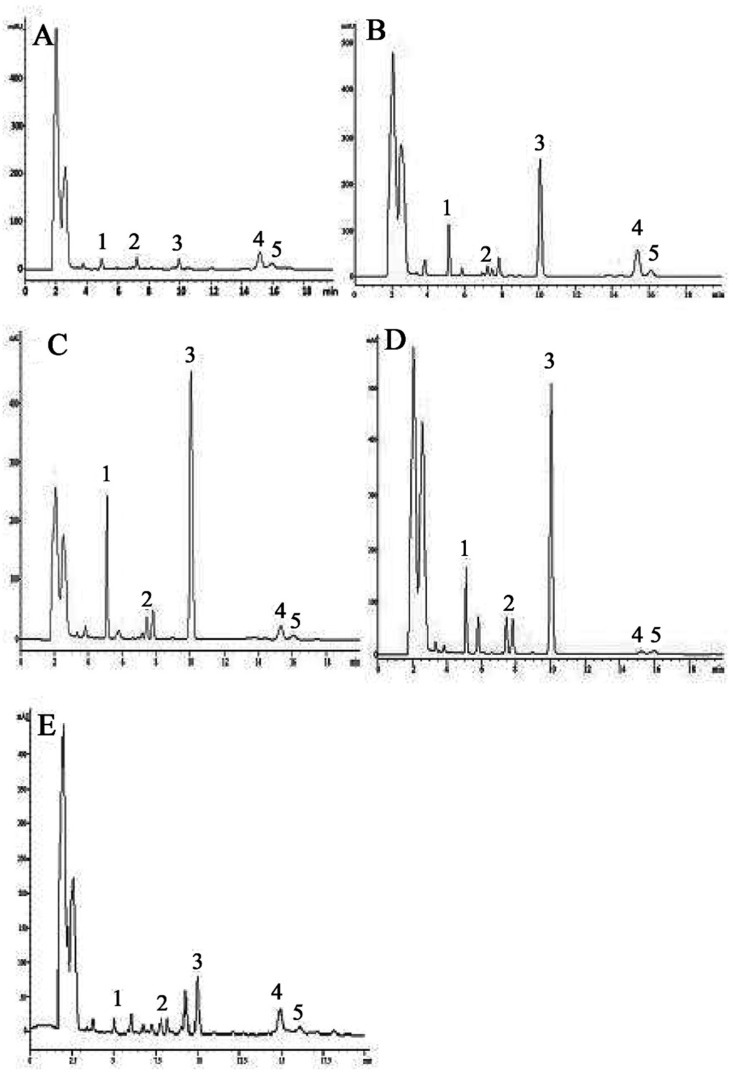
Representative HPLC chromatograms of different age *S. campanulatum* plants; (**A**) One year old; (**B**) Two years old; (**C**) Five years old; (**D**) Seven years old and (**E**) Ten years old.

The representative chromatograms (Figure 9) and data on flavanones, chalcone and triterpenoids in *S. campanulatum* plants of five different age is presented in Table 3. It was revealed from data that compounds (**1**–**5**) ranged from 22.24% to 72.97% in five different age plants (**A**–**E**)*.* It was observed that the age of the *S. campanulatum* plant generally has a very prominent impact on production of high valued secondary metabolites. Developed HPLC method is suitable for quantification of compounds (**1**–**5**) in standard mixtures and in plant extracts. Therefore, it can be concluded that use of developed method can save time, money and it can be used for routine analysis of compounds (**1**–**5**) in herbal laboratories with high accuracy and reproducibility.

**Figure 10:**
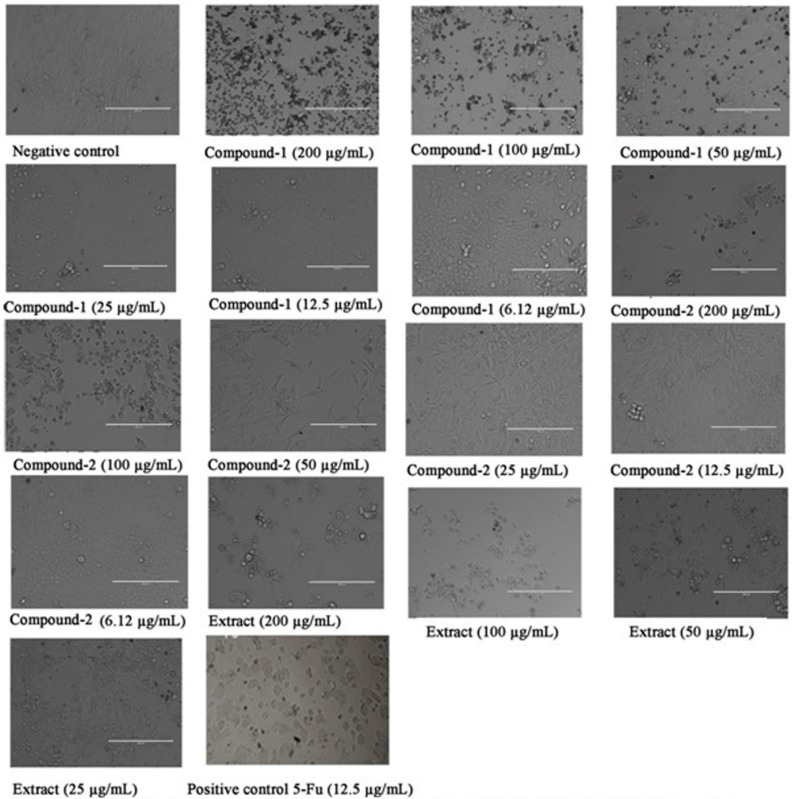
Dose dependent anti-proliferative effect of (2*S*)-7-Hydroxy-5-methoxy-6,8-dimethyl flavanone (compound-**1**), (*S*)-5,7-dihydroxy-6,8-dimethyl flavanone (compound-**2**) and *S. campanulatum* ethanolic extract on HCT 116 cell lines was assessed by MTT-assay.

Inhibition of Human Colorectal Carcinoma (HCT 116) cells proliferation by ethanolic extract of *S. campanulatum* and compounds (**1** and **2**) was performed. In correspondence to Figure 10, ethanolic extract of *S. campanulatum* and compounds (**1** and **2**) inhibited the proliferation on HCT 116 in dose-dependent manner. The median inhibitory concentrations (IC_50s_) for ethanolic extract of *S. campanulatum* and compounds (**1** and **2**) were then calculated with the accurate precision from the dose response curves. These were found to be anti-proliferative on the human colon cancer (HCT 116) cell line with IC_50_ 93.4, 67.6 and 132.9 µg·mL^−1^, respectively.

## 3. Experimental Section

### 3.1. Plant Material

The green leaves of *S. campanulatum* shrub of five different ages (1, 2, 5, 7 and 10 years old) were collected in June 2014, from the main campus of University Sains Malaysia (USM), Penang, Malaysia. The age of the shrub was determined based on the records of date of plantation by the Herbarium of School of Biological Sciences. The plant was authenticated by the Herbarium of School of Biological Sciences, USM, where a voucher specimen was deposited (Ref. No. 11047). The samples were labeled as Plant**-A** (One year old), Plant**-B** (2 years old), Plant**-C** (5 years old), Plant**-D** (7 years old) and Plant**-E** (10 years old).

### 3.2. Chemicals and Reagents

All solvents were used of HPLC-grade and analytical grade reagents, MTT (3-(4,5-dimethylthiazol-2-yl)-2,5 diphenyl tetrazolium bromide), 5-Fluorouracil 99% HPLC grade, phosphate-buffered saline (PBS), DMSO (dimethyl sulfoxide) were purchased from Sigma-Aldrich, Munich, Germany. Penicillin/streptomycin, fetal bovine serum and trypsin were purchased from Gibco, Life technology, Grand Island, NY, USA.

### 3.3. Cell Culture and Cell Lines

Human colorectal carcinoma (HCT 116), obtained from American Type Culture Collection (ATCC), Rockville, MD, USA. Cells were cultured in RPMI-1640 (Invitrogen, Gibco, Waltham, MA, USA) medium containing l-glutamine at 5% CO_2_-humidifed atmosphere at 37 °C growth medium supplemented with 10% heat-inactivated fetal bovine serum (HIFBS) and 1% penicillin/streptomycin (PS).

### 3.4. Extraction

The green leaves of *S. campanulatum* (**A**–**E**) were washed under tap water and dried at 45 °C in oven for 2–3 days and ground to fine powder using electric grinder (Retsch, Deutschland, Germany). The powdered leaves (250 g) were separately extracted by Soxhlet with a mixture of *n*-hexane:methanol (1:1) for 48 h. The extracts were filtered using Whatmann filter paper No. 1 and the filtrates were evaporated to dryness at 40 °C by using rotary evaporator (Buchi, New Castle, PA, USA). The crude extracts were further kept for 12 h at 45 °C in oven to ensure complete dryness. Crude extract of plant **C** (5 years old) was selected on the basis of high contents of compounds (**1**, **2** and **3**) for isolation and the other four plant samples were used for quantitative analysis of the marker compounds.

### 3.5. Isolation of Compounds (**1**–**4**)

The crude *n*-hexane:methanol (1:1) extract (20 g) was ground into fine powder using a mortar and pestle. Subsequently, the powdered material was subjected to flash column chromatography under vacuum as the following: column dimensions were 10 × 7 cm, the stationary consisted of silica gel (0.063–0.200 mm, 230–400 mesh). Elution was started with *n*-hexane, and continued with a mixture of *n*-hexane:ethyl acetate with decreasing ratio of *n*-hexane (92.5%, 60%, 30%, 15% and 0%). The volume of the eluent in each cycle was 250 mL, and a total of 19-fractions were obtained. The solvent was evaporated at room temperature in fume hood for 3–4 days. Compound (**1**) was obtained (200 mg) as a light green solid. Compounds **2** (100 mg) and **3** (400 mg) were obtained a yellow solid (100 mg). Compound **4** was obtained as a white solid (600 mg). All isolated compounds were studied further.

### 3.6. Crystallization

Compound (**1**) was dissolved in 2 mL acetone, compound (**2**) in 2 mL *n*-hexane, compound (**3**) in 2 mL *n*-hexane and 0.2 mL methanol, and compound (**4**) was dissolved in 2 mL methanol, filtered through a 0.45-μm filter and allowed to evaporate at room temperature. Compound (**1**) produced needle type white crystals and compound (**2**) produced yellow crystals. Purity of crystals of compounds (**1** and **2**) was confirmed by HPLC, UV, FTIR, NMR and X-ray crystallography. Compound (**3**) produced needle type yellow crystals and compound (**4**) produced white crystals, while the purity of crystals of compounds (**3** and **4**) were analyzed as discussed [3,5].

### 3.7. Characterization of Compounds

#### 3.7.1. UV-Visible Spectroscopy

All compounds (**1**–**5**) were dissolved in methanol and scanned from 500–200 nm for maximum absorbance using Double beam UV/Visible spectrometer (Lambda 25, Perkin-Elmer, Waltham, MA, USA) with dual silica 1 cm cuvettes controlled by UVWinLab 25 software (Waltham, MA, USA).

#### 3.7.2. FTIR Spectroscopy

The crystals of compounds (**1**–**4**) and compound (**5**) as powder were characterized by FTIR spectrophotometer (Perkin Elmer Spectrum one FTIR spectrometer, USA) using potassium bromide (KBr) disc method. The IR spectrum was scanned at infrared region of 400–4000 cm^−1^. Furthermore, *n*-hexane:methanol (1:1) crude extracts were prepared from *S. campanulatum* at five different ages (**A**–**E**) and scanned using FTIR spectroscopy.

#### 3.7.3. NMR Spectroscopy

Compounds (**1** and **2**) were analyzed by 2D-NMR spectra (FT-NMR spectrometer, Bruker 500 MHz) in deuterated methanol (MeOD). The NMR peaks were labeled as singlet (s), doublet (d), triplet (t) and multiplet (m) chemical shifts were referenced with respect to solvent signals.

#### 3.7.4. X-ray Crystallography

The single crystal of compounds (**1** and **2**) obtained were analyzed by Bruker SMART APEX2 CCD area detector diffractometer. The molecular graphics were constructed by Mercury 3.1 Development (Build RC5) software (Cambridge, UK).

#### 3.7.5. LC-EIMS Analysis

Mass spectra (*n* = 5) of compounds (**1** and **2**) were determined using a Micro QTof ESI Mass Spectrometer (Waters) coupled with LCMS Xevo G2 QTof, USA. The compounds (**1** and **2**) were prepared separately in MS grade methanol and 2 µL of each compound was injected through UPLC into ESI probe. The MS conditions: column; ACQUITY UPLC BEH C18, 1.7 µ (Waters, Europe/Dublin, Ireland), column dimension 2.1 × 50 mm, part number: 186002350, mobile phase; double distilled deionized water and acetonitrile both with 0.1% formic acid (100 µL·100 mL^−1^) at a flow rate 0.3 mL·min^−1^ positive ion mode; gas (N_2_); temperature, 350 °C; flow rate, 10 L·min^−1^, nebulizer pressure, 15 psi; sample infusion flow rate 20 µL·min^−1^, HV voltage, 4.0 kV; octopole RF amplitude, 150 Vpp; skim 1 voltage, −38.8 V; skim 2 voltage, −6.0 V; cap (KV) 3 V; sampling cone 40, extraction cone 4, and scan range, *m*/*z* 100–1000 units. Acquired mass spectra represented the average of *n* = 5 spectra.

#### 3.7.6. Preparation of Stock Solutions for HPLC Analysis

Stock solutions of all compounds (**1**–**5**) and extracts of *S. campanulatum* plant (**A**–**E**) were prepared at the concentration of 1 mg·mL^−1^ in methanol, filtered using 0.45 µm filter and stored at 25 °C. The stock solution of compounds (**1**–**5**) was further diluted to the concentration range 250–0.24 µg·mL^−1^.

### 3.8. HPLC Method Validation

The developed method was validated for the following parameters: linearity, precision, accuracy, selectivity, robustness, limit of quantification (LOQ), limit of detection (LOD) and system suitability [29,30].

#### 3.8.1. Selection of Detection Wavelength

The UV spectrum of diluted solutions of compounds (**1**–**5**) was recorded using UV spectrophotometer. It was observed that compounds (**1**–**3**) have shown absorbance at 330 nm but no absorbance was shown by (**4** and **5**). So, λ_max_ 210 nm was selected as best for all compounds (**1**–**5**).

#### 3.8.2. Linearity

Linearity was studied in the range 250–0.24 μg·mL^−1^ of the standard compounds (**1**–**5**).Ten microliter of each solution was injected into the HPLC system, and the linearity for each compound (**1**–**5**) was calculated by plotting peak area versus concentration. The calibration curves of compounds (**1**–**5**) were linear with relatively wide range of concentrations 0.78–200 µg·mL^−1^ for (**1**–**3**), 12.5–200 (**4**) and 3.1–200 µg·mL^−1^ for (**5**). All compounds (**1**–**5**) showed good linear regressions with high correlation coefficient values (*r*^2^ ≤ 0.999) between peak area (y) and concentrations of each compound (x, µg·mL^−1^).

#### 3.8.3. Accuracy

Accuracy of developed method was determined at three concentration levels: 250, 125 and 62.5 μg·mL^−1^ (*n* = 3), and the percentage recovery was calculated. Peak areas of the compounds (**1**–**5**) in plant (**A**–**C**) extracts (W), reference compounds (Z) and their combinations (X) were recorded. Percentage recovery was calculated using the formula: % Recovery = (X − W)/Z × 100 [30] and the results are presented as mean ± SD (*n* = 3).

#### 3.8.4. Precision

The intra-day and inter-day precisions of developed method were evaluated at four concentrations: 250, 125, 62.5 and 31.25 µg·mL^−1^ (*n* = 3). The result was calculated as % RSD. The LOQ and LOD were determined based on the standard deviation of the response and the slope of the constructed calibration curves (*n* = 3) as described [29,30].

#### 3.8.5. Robustness

The robustness of the developed method was performed by introducing small changes in the optimized HPLC conditions such as wavelength (±5 nm), percentage of acetonitrile in the mobile phase (±10%), flow rate (±0.1 mL·min^−1^) and column temperature (±5 °C).

#### 3.8.6. Specificity

The specificity of developed method was evaluated by observing interference of chemically related compound, cardamonin (compound 6). The test result was compared with that obtained for standard compounds (**1**–**5**).

#### 3.8.7. Reproducibility

The reproducibility of developed method was investigated by determining precision by another analyst on a different instrument and laboratory.

### 3.9. Quantification of Compounds (**1**–**5**) in Mixture and Extracts

HPLC analysis was performed using Agilent HPLC 1260 system, on ZORBAX Eclipse Plus Phenyl Hexyl column (4.6 × 250 mm, 5 µ). The mobile phase consisted of acetonitrile: 0.1% *o*-phosphoric acid. The elution program was isocratic at 60:40 for 20 min, at 1 mL·min^−1^. A sample of 10 µL (250–0.24 µg·mL^−1^) was injected at λ_max_ 210 nm. The purity of compounds (**1**–**5**) was assured from chromatograms, and the plant extracts were analyzed for the presence of all compounds (**1**–**5**). Linear regression equations of the calibration curves were applied to calculate the concentration of the marker compounds in *S. campanulatum* extracts. The results were calculated as % *w*/*w* using the formula: (Calculated concentration/Loaded concentration) × 100% [3].

### 3.10. Cells Proliferation Assay Using MTT

The assay was carried out using the method described by [31] with minor modification. Cells were seeded at density of 1–1.5 × 10^5^ mL^−1^ in 96 wells flat-bottomed plates, after 24 h the extracts or positive control were added to each well within 100 µL media at different concentration and free culture media used as a blank. DMSO was used as a negative control and 5-FU as a positive control. All tested samples were performed in six serial concentrations as follow; 200, 100, 50, 25, 12.5 and 6.125 µg·mL^−1^. The plates were incubated at 37 °C incubator under 5% CO_2_. After 48 h incubation, 20 µL of MTT reagent solution (5 mg·mL^−1^ in PBS) was added to each well as an indicator of cell viability. The reading of the colorimetric assay was taken using micro plate reader Infinit pro200 (Tecan, Männedorf, Switzerland) at a wavelength of 570 and 620 nm. The percentage of inhibition was calculated according to the equation: Cell inhibition % = (1 − (A Sample − A Blank)/(A Control − A Blank)) × 100.

## 4. Conclusions

It is concluded from the present study that **1**–**5** year old plants produced high amount of compounds (**1**–**5**) as secondary metabolites in selected five differently aged plants of the same specie. Newly developed HPLC method is accurate, precise, reproducible, and specific. In the present study, the isolation of compounds (**1** and **2**) from dried leaves of *S. campanulatum* with their purification, detailed chemical structural characterization and anti-proliferative activity is reported. It has been observed that both compounds (**1** and **2**) and extract of *S. campanulatum* possess strong anti-proliferative activity against Human colorectal carcinoma (HCT 116) cells.

## Supplementary Materials

Compounds (**1** and **2**) X-ray crystallographic data of the structures have been deposited with the Cambridge Crystallographic Data Center, CCDC1026578 and 1007509, respectively. This data of both compounds can be obtained free of charge from CCDC via www.ccdc.cam.ac.uk/data_request/cif.

Supplementary materials can be accessed at: http://www.mdpi.com/1420-3049/20/08/14212/s1.
