# Supplementary material for: Isolation, Characterization, Crystal Structure Elucidation of Two Flavanones and Simultaneous RP-HPLC Determination of Five Major Compounds from Syzygium campanulatum Korth
Source: Molecules. 2015 Aug 4;20(8):14212–33. doi: 10.3390/molecules200814212 (PMC6331876; doi:10.3390/molecules200814212)
Supplement: Supplementary file 1 [file molecules-20-14212-s001.pdf]

Supplementary Materials

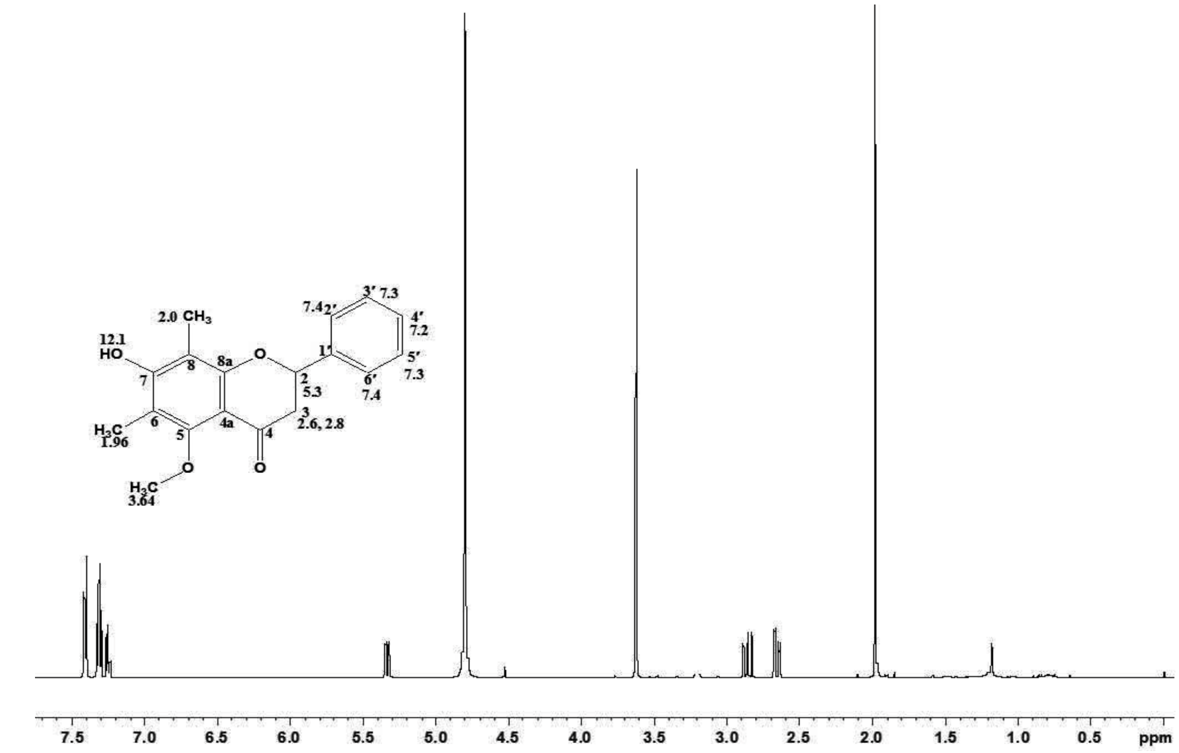

Figure S1. ^{1}H-NMR spectra compound-**1**.

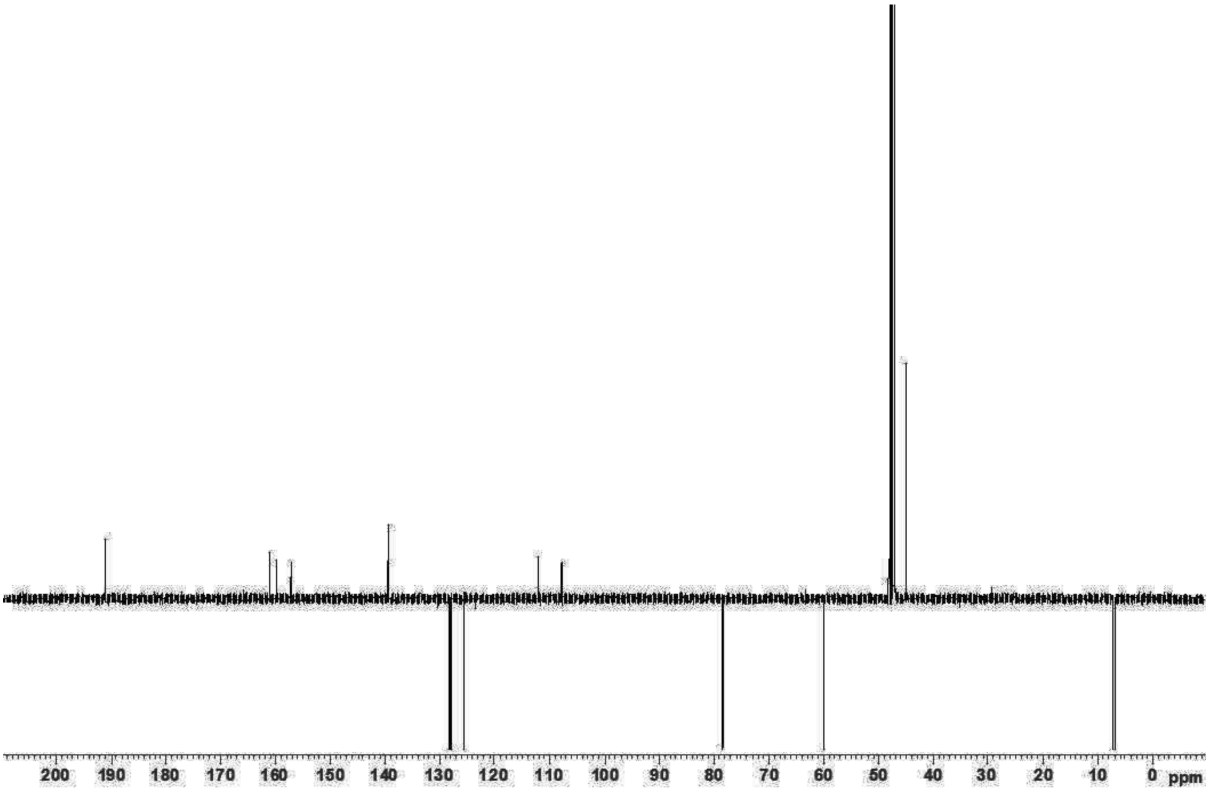

Figure S2. ^{13}C-DEPTQ-NMR spectra compound-**1**.

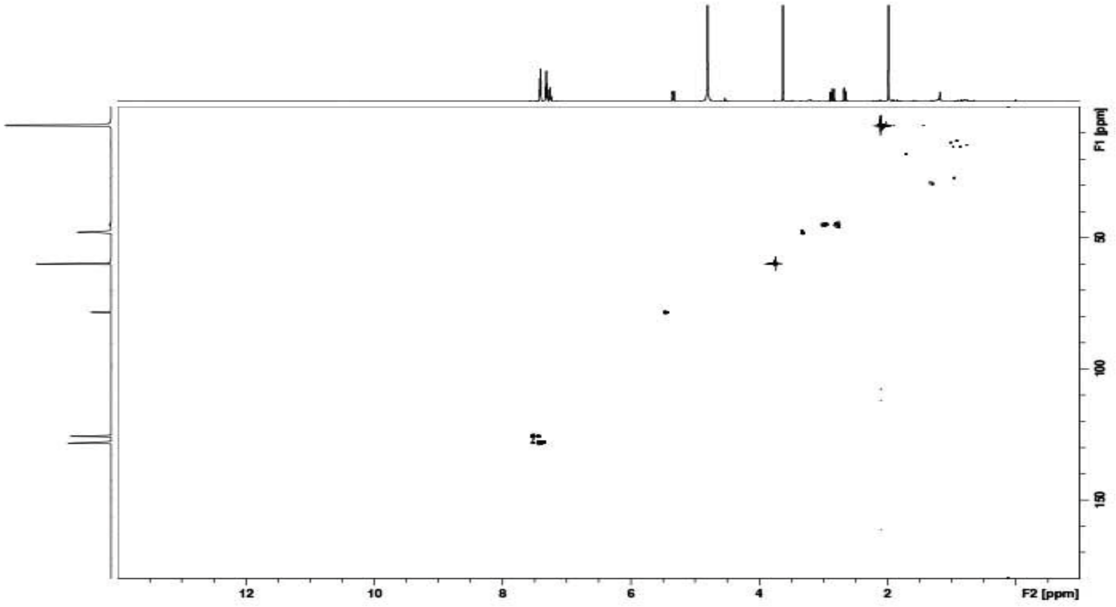

Figure S3. HSQC-NMR spectra compound-**1**.

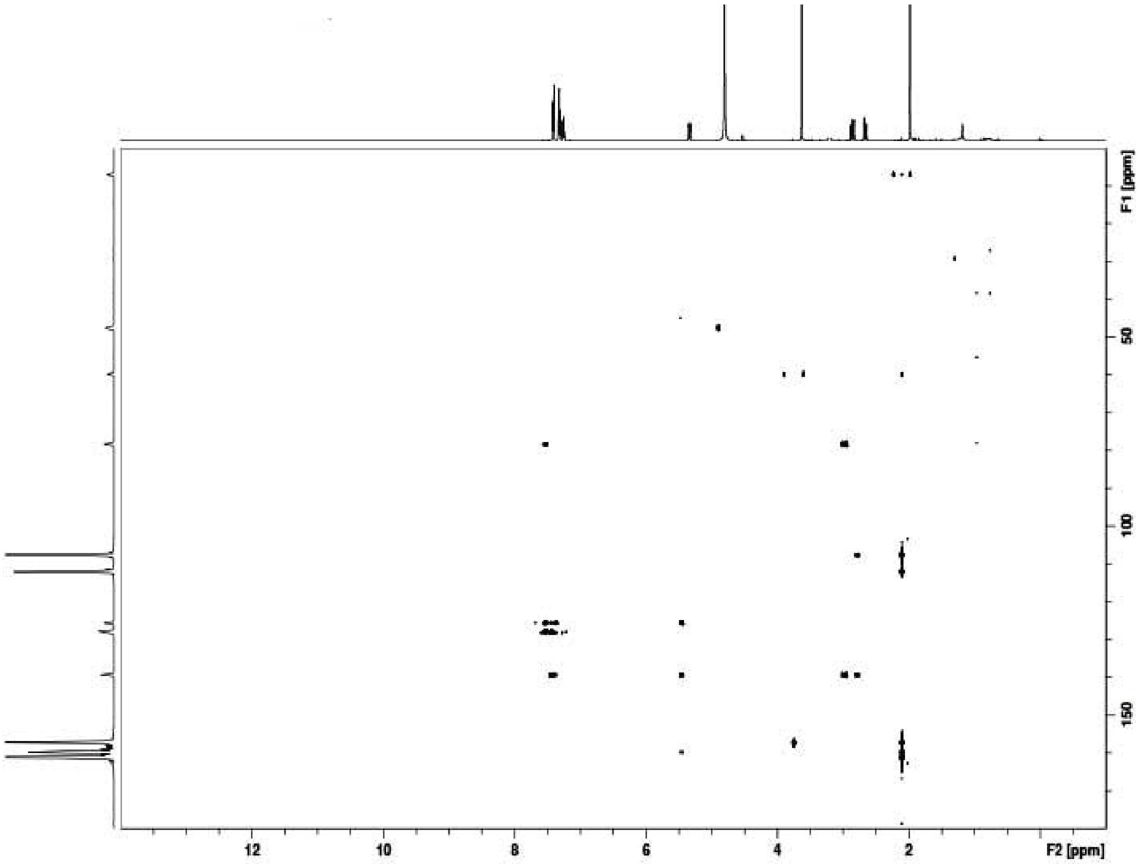

Figure S4. HMBC-NMR spectra compound-**1**.

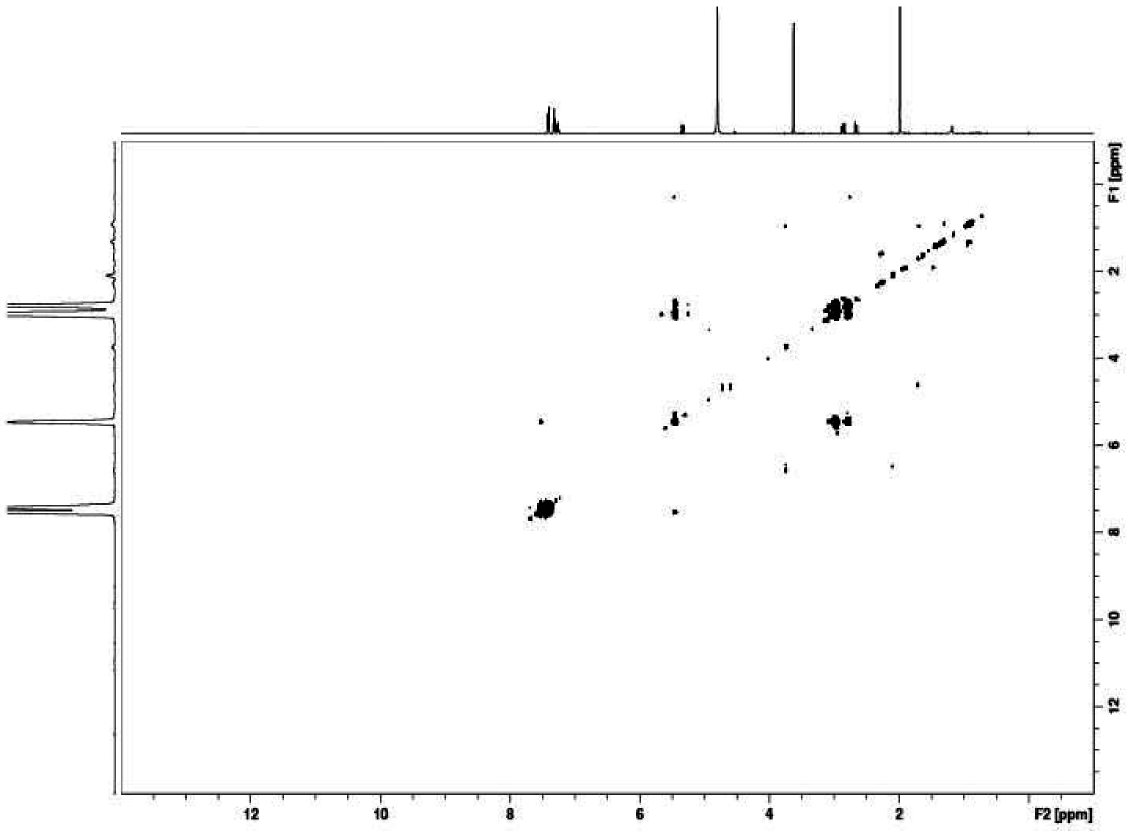

Figure S5. 2D-COSY-NMR spectra compound-**1**.

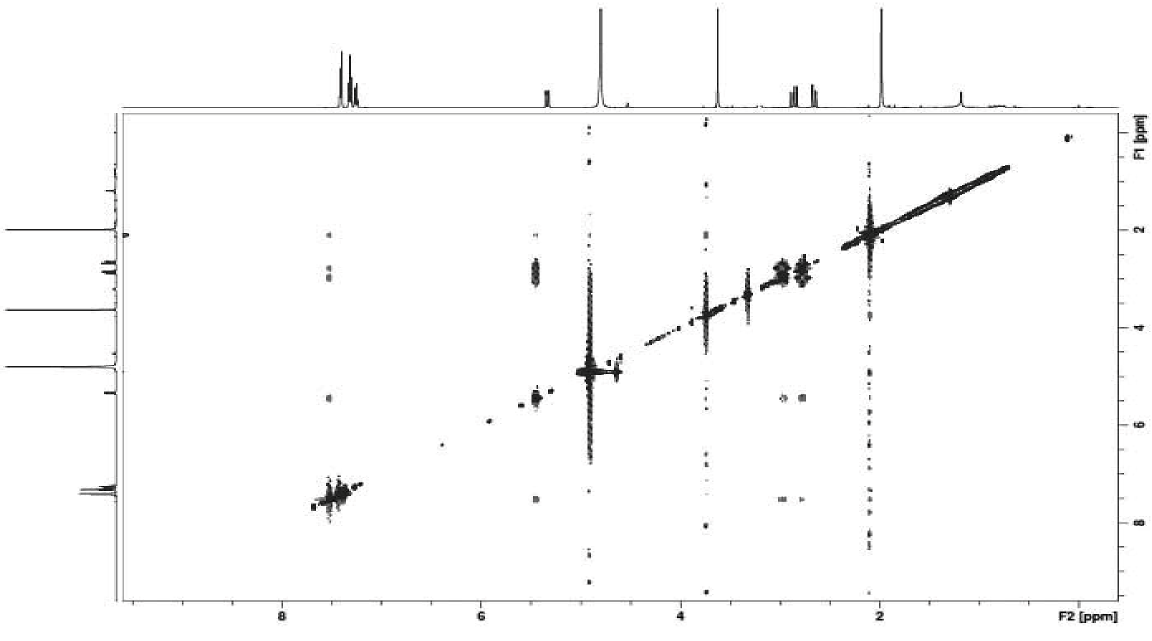

Figure S6. 2D-NOESY-NMR spectra compound-**1**.

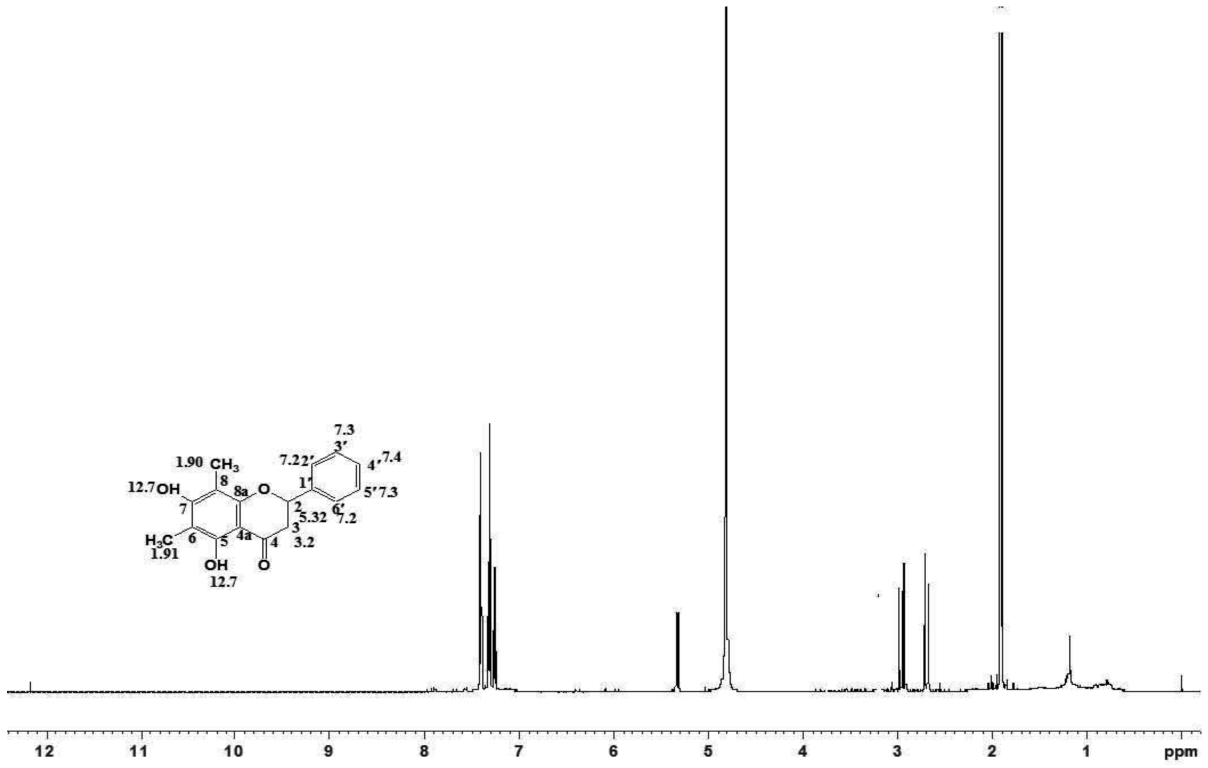

Figure S7. ^{1}H-NMR spectra compound-**2**.

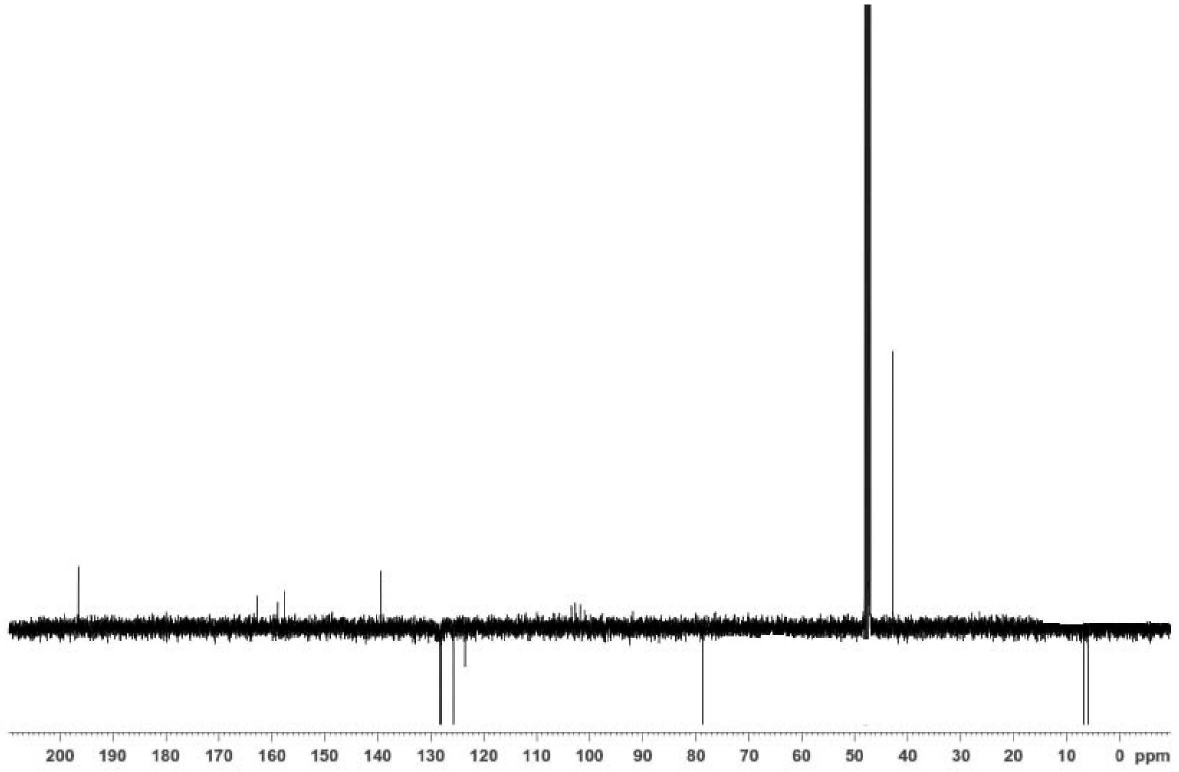

Figure S8. ^{13}C-DEPTQ-NMR spectra compound-**2**.

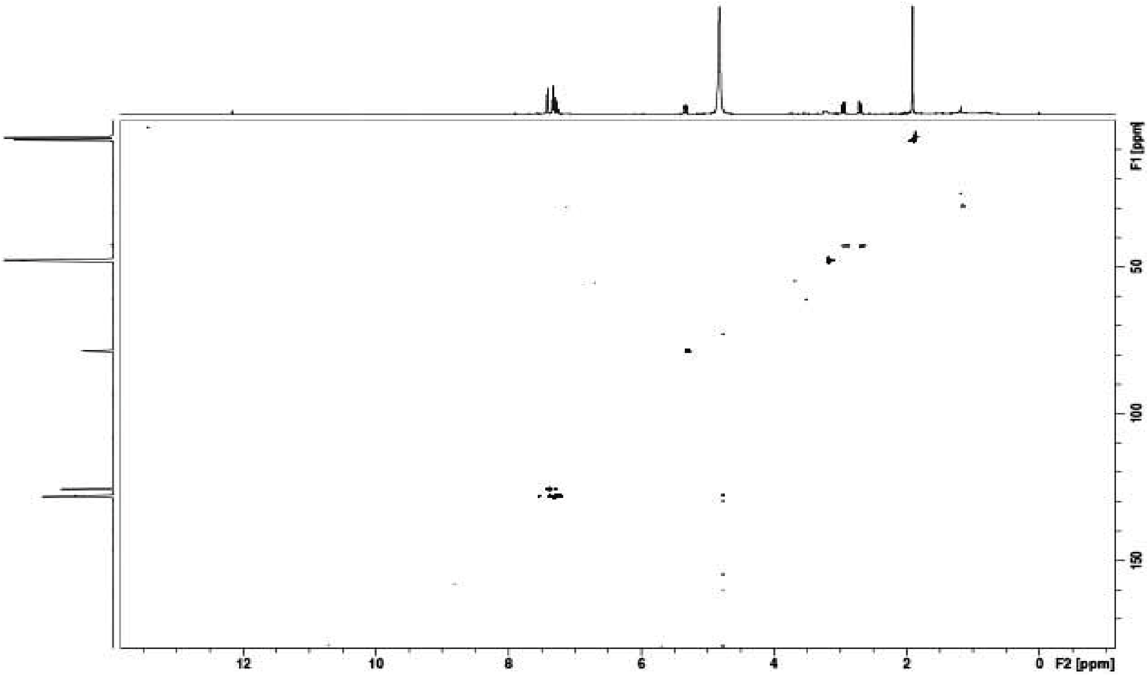

Figure S9. HSQC-NMR spectra compound-**2**.

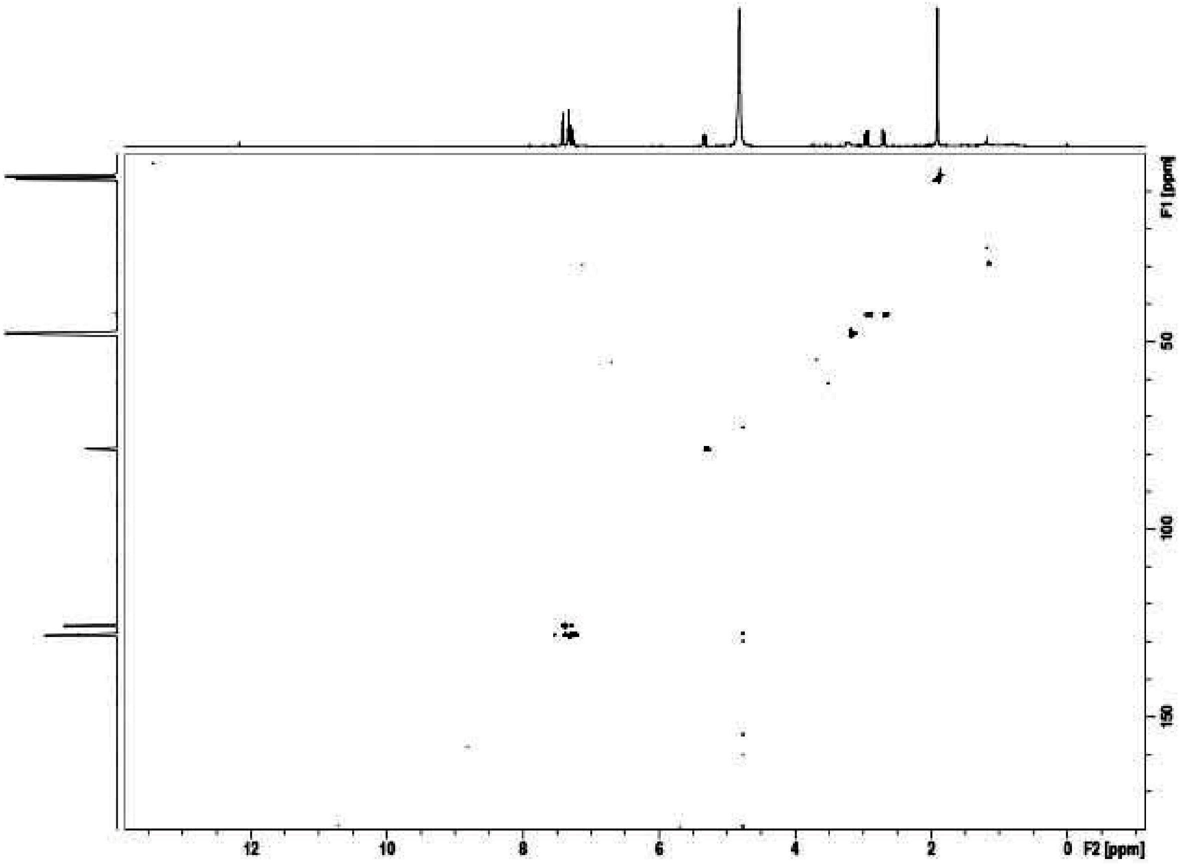

Figure S10. HMBC-NMR spectra compound-**2**.

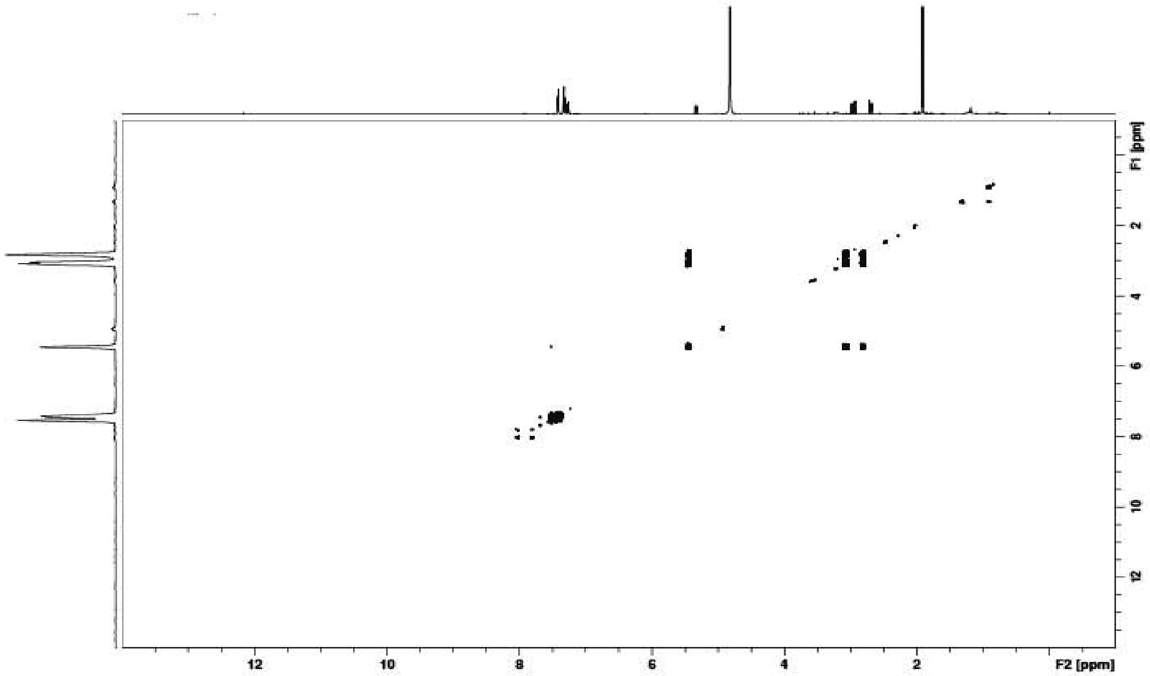

Figure S11. 2D-COSY-NMR spectra compound-**2**.

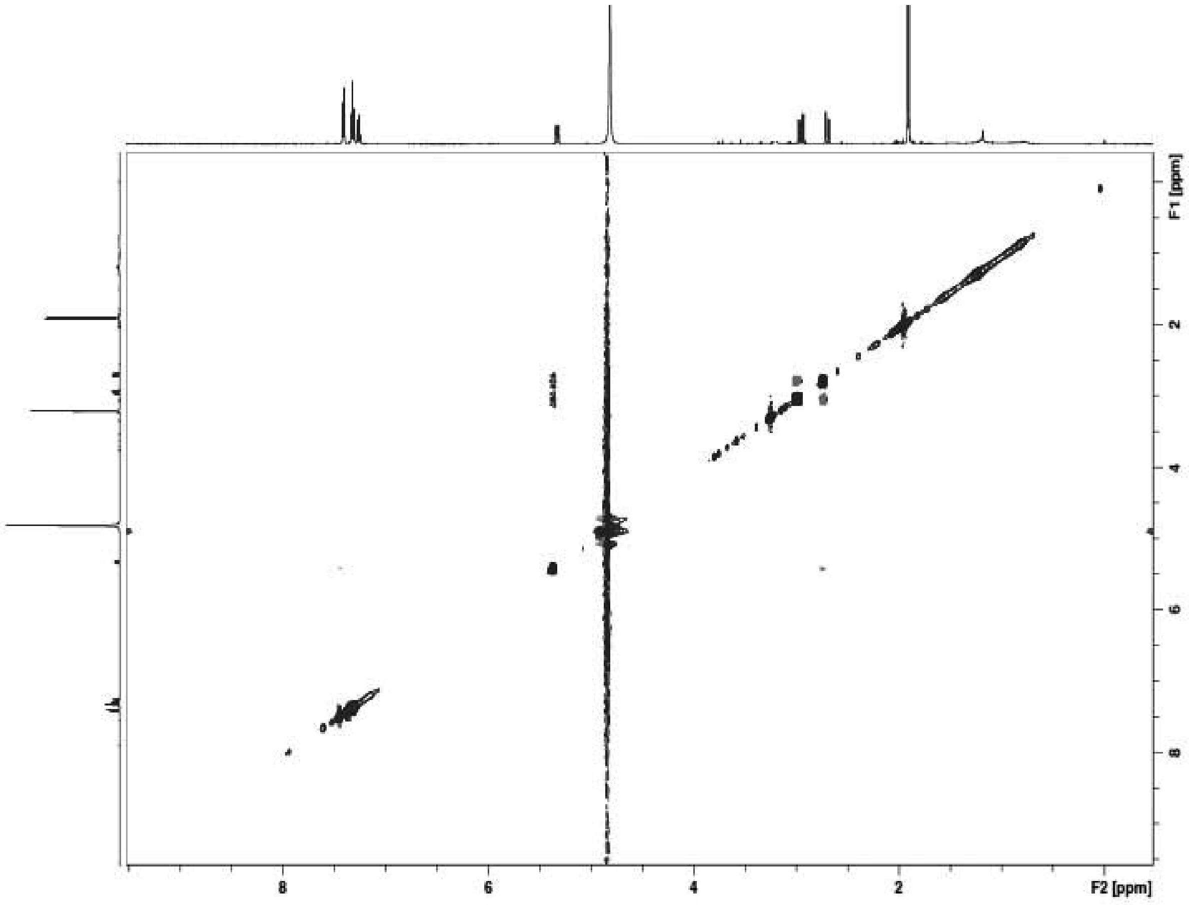

FigureS12. 2D-NOESY-NMR spectra compound-**2**.

Supplementary Material of Compound 1: (2*S*)-7-Hydroxy-5-methoxy-6,8-dimethyl flavanone.

Table S1. Crystal Data and Details of the Structure Determination for: mo_f16_0m P 21/c R = 0.08.

Table S2. Final Coordinates and Equivalent Isotropic Displacement Parameters of the non-Hydrogen atoms for: mo_f16_0m P 21/c, R = 0.08.

Table S3. Hydrogen Atom Positions and Isotropic Displacement Parameters for: mo_f16_0m P 21/c, R = 0.08.

Table S4. (An) isotropic displacement parameters for: mo_f16_0m P 21/c, R = 0.08.

Table S5. Bond distances (angstrom) for: mo_f16_0m P 21/c, R = 0.08.

Table S6. Bond angles (degrees) for: mo_f16_0m P 21/c, R = 0.08.

Table S7. Torsion angles (degrees) for: mo_f16_0m P 21/c, R = 0.08.

Table S8. Contact distances (angstrom) for: mo_f16_0m P 21/c, R = 0.08.

Table S9. Hydrogen bonds (angstrom, Deg) for: mo_f16_0m P 21/c, R = 0.08.

Table S1. Crystal data and details of the structure determination for: mo_f16_0m P 21/c, R = 0.08.

Crystal Data	
Formula	C18H18O4
Formula Weight	298.32
Crystal System	Monoclinic
Space group	P21/c (No. 14)
a, b, c [Angstrom]	12.7683 (14), 17.2730 (15), 7.2728 (7)
alpha, beta, gamma [deg]	90, 105.966 (3), 90
V [$Å^3$]	1542.1 (3)
Z	4
D (calc) [g/cm^3]	1.285
Mu (MoKa) [/mm]	0.090
F(000)	632
Crystal Size [mm]	0.12 × 0.17 × 0.48
**Data Collection**	
Temperature (K)	294
Radiation [Angstrom]	MoKa 0.71073
Theta Min-Max [Deg]	1.7, 27.5
Dataset	−16:16; −22:22; −8:9
Tot., Uniq. Data, R (int)	15650, 3514, 0.042
Observed data [I > 2.0 sigma(I)]	2558
**Refinement**	
Nref, Npar	3514, 274
R, wR2, S	0.0775, 0.2521, 0.98
w = ^2^(FO^2^) + (0.1690P)^2^ + 0.7080P]	WHERE P = (FO^2^ + 2FC^2^)/3′
Max. and Av. Shift/Error	0.01, 0.00
Min. and Max. Resd. Dens. [e/Ang^3]	−0.54, 0.90

Table S2. Final coordinates and equivalent isotropic displacement parameters of the non-hydrogen atoms for: mo_f16_0m P 21/c, R = 0.08.

Atom	X	Y	Z	U (eq) [Ang^2]
O1	0.30825(10)	0.43580(7)	0.1711(2)	0.0435(4)
O2	0.60772(12)	0.60656(8)	0.3139(2)	0.0459(4)
O3	0.67455(11)	0.34310(8)	0.2342(2)	0.0437(4)
O4	0.49424(13)	0.24744(8)	0.1414(3)	0.0561(5)
C1	0.41789(14)	0.44505(10)	0.2053(2)	0.0330(4)
C2	0.45384(14)	0.52132(9)	0.2394(2)	0.0329(4)
C3	0.56513(15)	0.53422(10)	0.2775(2)	0.0339(4)
C4	0.64061(14)	0.47483(10)	0.2833(3)	0.0365(5)
C5	0.60129(14)	0.40020(10)	0.2448(3)	0.0350(5)
C6	0.48901(14)	0.38256(10)	0.2049(3)	0.0343(4)
C7	0.44255(15)	0.30561(10)	0.1554(3)	0.0396(5)
* C8	0.3166(3)	0.3032(2)	0.0908(6)	0.0401(10)
* C9	0.2728(2)	0.36046(15)	0.2137(5)	0.0297(7)
* C10	0.1483(4)	0.3606(3)	0.1671(10)	0.0630(8)
* C11	0.0951(4)	0.3170(4)	0.2485(11)	0.0924(16)
* C12	−0.0175(5)	0.3177(5)	0.2100(12)	0.0997(16)
* C13	−0.0771(5)	0.3614(5)	0.0713(13)	0.0821(15)
* C14	−0.0291(5)	0.4062(4)	−0.0254(13)	0.1000(16)
* C15	0.0845(4)	0.4070(4)	0.0136(11)	0.0914(16)
C16	0.37462(16)	0.58557(11)	0.2329(3)	0.0434(5)
C17	0.76017(17)	0.49194(13)	0.3316(4)	0.0559(7)
C18	0.70897(19)	0.29479(13)	0.3986(4)	0.0565(7)
* C12X	0.0023(6)	0.3529(5)	0.2708(16)	0.0876(17)
* C13X	−0.0702(8)	0.3561(7)	0.1000(19)	0.0850(17)
* C14X	−0.0348(6)	0.3637(5)	−0.0614(16)	0.0901(17)
* C8X	0.3295(5)	0.2972(4)	0.1577(11)	0.0536(14)
C9X	0.2639(5)	0.3626(4)	0.0862(13)	0.0691(14)
* C10X	0.1494(6)	0.3628(4)	0.1204(13)	0.0630(8)
* C11X	0.1090(6)	0.3523(4)	0.2953(15)	0.0803(16)
* C15X	0.0784(6)	0.3650(5)	−0.0462(15)	0.0840(16)
C9X	0.2639(5)	0.3626(4)	0.0862(13)	0.0691(14)

U (eq) = 1/3 of the trace of the orthogonalized U Tensor Starred Atom sites have a S.O.F less than 1.0.
* Note: Carbon atom showed statistical conformational disorders.

Table S3. Hydrogen Atom Positions and Isotropic Displacement Parameters for: mo_f16_0m P 21/c, R = 0.08.

Atom	X	Y	Z	U (iso) [Ang^2]
* H8A	0.29040	0.31730	−0.04320	0.0480
* H8B	0.29140	0.25120	0.10630	0.0480
H1O2	0.555(3)	0.6390(18)	0.317(4)	0.073(9)
H16C	0.40070	0.61820	0.34280	0.0650
H17A	0.80060	0.44520	0.37250	0.0840
H17B	0.77870	0.52950	0.43260	0.0840
H17C	0.77780	0.51200	0.22060	0.0840
H18A	0.74950	0.25170	0.37020	0.0850
H18B	0.64630	0.27600	0.43370	0.0850
H18C	0.75430	0.32400	0.50270	0.0850
* H9A	0.30440	0.34870	0.34950	0.0360
* H11A	0.13390	0.28230	0.33990	0.1110
* H12A	−0.05130	0.28720	0.28230	0.1200
* H13A	−0.15270	0.36030	0.04270	0.0990
* H14A	−0.07080	0.43790	−0.12120	0.1200
* H15A	0.11820	0.43740	−0.05920	0.1090
H16A	0.30510	0.56430	0.23290	0.0650
H16B	0.36710	0.61550	0.11870	0.0650
* H8XA	0.29870	0.25230	0.08190	0.0640
* H8XB	0.32750	0.28760	0.28810	0.0640
* H9XA	0.25460	0.36570	−0.05190	0.0830
* H11B	0.15730	0.34570	0.41590	0.0960
* H12B	−0.02330	0.35090	0.37870	0.1060
* H13B	−0.14430	0.35330	0.08980	0.1020
* H14B	−0.08520	0.36800	−0.18070	0.1080
* H15B	0.10180	0.36740	−0.15630	0.1010

The Temperature Factor has the Form of Exp (−T) Where T = 8 * (Pi ** 2) * U * (Sin (Theta)/Lambda) ** 2 for Isotropic Atoms. * Note: Hydrogen atom showed statistical conformational disorders.

Table S4. (An) isotropic displacement parameters for: mo_f16_0m P 21/c, R = 0.08.

Atom	U(1,1) or U(2,2)	U(3,3)	U(2,3)	U(1,3)	U(1,2)
O1	0.0326(6) 0.0276(6)	0.0677(9)	−0.0014(6)	0.0096(6)	0.0001(5)
O2	0.0450(7) 0.0267(6)	0.0658(9)	−0.0037(6)	0.0149(6)	−0.0056(5)
O3	0.0430(6) 0.0335(6)	0.0584(8)	0.0038(6)	0.0205(6)	0.0096(5)
O4	0.0520(8) 0.0264(6)	0.0907(11)	−0.0075(7)	0.0212(8)	0.0027(6)
C1	0.0344(8) 0.0265(7)	0.0376(8)	0.0015(6)	0.0089(7)	0.0004(6)
C2	0.0383(8) 0.0257(7)	0.0354(8)	0.0014(6)	0.0111(6)	0.0022(6)
C3	0.0402(8) 0.0256(7)	0.0359(8)	−0.0007(6)	0.0107(7)	−0.0031(6)
C4	0.0362(8) 0.0313(8)	0.0426(9)	0.0039(7)	0.0118(7)	0.0012(7)
C5	0.0377(8) 0.0289(8)	0.0409(8)	0.0027(7)	0.0149(7)	0.0047(6)
C6	0.0378(8) 0.0249(7)	0.0409(8)	0.0003(7)	0.0118(7)	0.0005(6)
C7	0.0426(9) 0.0262(8)	0.0495(10)	−0.0022(7)	0.0121(8)	0.0002(7)
C8	0.0376 (15) 0.0266 (14)	0.053(2)	−0.0081(14)	0.0072(14)	−0.0038(12)
C9	0.0294(12) 0.0214 (11)	0.0376(13)	−0.0002(11)	0.0081(11)	−0.0021(9)
C10	0.0395(10) 0.0376 (10)	0.106(2)	0.0000(14)	0.0102(13)	−0.0024(8)
C11	0.056(2) 0.085(3)	0.130(3)	0.027(3)	0.015(2)	−0.008(2)
C12	0.055(2) 0.102(3)	0.141(3)	0.018(3)	0.025(2)	−0.015(2)
C13	0.0400 (19) 0.074(3)	0.128(3)	−0.012(3)	0.016(2)	−0.002(2)
C14	0.057(2) 0.093(3)	0.136(3)	0.023(3)	0.003(2)	0.005(2)
C15	0.055(2) 0.090(3)	0.124(3)	0.027(3)	0.016(2)	−0.003(2)
C16	0.0450 (9) 0.0266 (8)	0.0589(11)	−0.0033(8)	0.0149(8)	0.0028(7)
C17	0.0383(9) 0.0438(11)	0.0863(16)	0.0009(11)	0.0182(10)	−0.0042(8)
C18	0.0575(12) 0.0451(11)	0.0648(13)	0.0091(10)	0.0131(10)	0.0195(9)
C12X	0.057(3) 0.078(3)	0.126(3)	−0.016(3)	0.022(3)	0.004(3)
C13X	0.047(3) 0.077(3)	0.129(3)	−0.005(3)	0.021(3)	−0.002(3)
C14X	0.053(3) 0.084(3)	0.122(3)	−0.005(3)	0.005(3)	0.002(3)
C8X	0.047(2) 0.037(2)	0.074(3)	−0.006(2)	0.012(2)	−0.005(2)
C9X	0.056(2) 0.045(2)	0.099(3)	−0.006(3)	0.009(2)	−0.002(2)
C10X	0.0395(10) 0.0376(10)	0.106(2)	0.0000(14)	0.0102(13)	−0.0024(8)
C11X	0.051(2) 0.063(3)	0.119(3)	−0.015(3)	0.010(2)	0.004(2)
C15X	C15X 0.057(2) 0.072(3)	0.123(3)	0.004(3)	0.025(3)	0.001(3)

The Temperature Factor has the Form of Exp (−T) Where T = 8 * (Pi ** 2) * U * (Sin (Theta)/Lambda) ** 2 for Isotropic Atoms. T = 2 * (Pi ** 2) * Sumij (h (i) * h (j) * U (i,j) * Astar(i) * Astar (j)), for An Isotropic Atoms. Astar (i) are Reciprocal Axial Lengths and H (i) are the Reflection Indices.

Table S5. Bond distances (angstrom) for: mo_f16_0m P 21/c, R = 0.08.

Bond Distances (Angstrom)			
O1-C1	1.362(2)	C11X-C12X	1.325(12)
O1-C9	1.439(3)	C12-C13	1.321(12)
O1-C9X	1.452(7)	C12X-C13X	1.331(17)
O2-C3	1.359(2)	C13-C14	1.306(11)
O3-C5	1.376(2)	C13X-C14X	1.375(16)
O3-C18	1.425(3)	C14-C15	1.399(9)
O4-C7	1.222(2)	C14X-C15X	1.419(12)
O2-H1O2	0.88(4)	C8-H8A	0.9700
C1-C2	1.395(2)	C8-H8B	0.9700
C1-C6	1.411(3)	C8X-H8XA	0.9700
C2-C16	1.494(3)	C8X-H8XB	0.9700
C2-C3	1.389(3)	C9-H9A	0.9800
C3-C4	1.400(3)	C9X-H9XA	0.9800
C4-C17	1.499(3)	C11-H11A	0.9300
C4-C5	1.384(2)	C11X-H11B	0.9300
C5 -C6	1.415(3)	C12-H12A	0.9300
C6 -C7	1.460(3)	C12X-H12B	0.9300
C7-C8X	1.455(7)	C13-H13A	0.9300
C7-C8	1.547(5)	C13X-H13B	0.9300
C8-C9	1.538(5)	C14-H14A	0.9300
C8X-C9X	1.417(10)	C14X-H14B	0.9300
C9-C10	1.532(6)	C15-H15A	0.9300
C9X-C10X	1.549(11)	C15X-H15B	0.9300
C10-C11	1.265(9)	C16-H16A	0.9600
C10-C15	1.433(10)	C16-H16C	0.9600
C10X-C15X	1.299(13)	C16-H16B	0.9600
C10X-C11X	1.509(13)	C17-H17B	0.9600
C11-C12	1.387(9)	C17-H17C	0.9600
C17-H17A	0.9600	C18-H18C	0.9600
C18-H18B	0.9600	C18-H18A	0.9600

Table S6. Bond Angles (Degrees) for: mo_f16_0m P 21/c, R = 0.08.

**Bond Angles (Degrees)**			
C1-O1-C9	115.79(16)	C7-C8X-C9X	113.8(5)
C1-O1-C9X	116.2(3)	O1-C9-C8	105.7(2)
C5-O3-C18	115.49(17)	C8-C9-C10	113.2(3)
C3-O2-H1O2	109(2)	O1-C9-C10	108.6(3)
O1-C1-C2	114.50(15)	O1-C9X-C10X	101.8(5)
C2-C1-C6	122.85(17)	C8X-C9X-C10X	115.6(6)
O1-C1-C6	122.65(15)	O1-C9X-C8X	114.6(6)
C1-C2-C16	120.62(16)	C9-C10-C11	123.6(5)
C3-C2-C16	122.27(15)	C11-C10-C15	115.7(6)
C1-C2-C3	117.11(16)	C9-C10-C15	120.4(5)
O2-C3-C4	115.62(17)	C11X-C10X-C15X	118.5(8)
C2 -C3-C4	123.12(16)	C9X-C10X-C15X	107.3(8)
O2-C3-C2	121.25(16)	C9X-C10X-C11X	133.8(8)
C3-C4-C17	120.72(17)	C10-C11-C12	124.1(7)
C5-C4-C17	121.37(17)	C10X-C11X-C12X	117.6(9)
C3-C4-C5	117.90(17)	C11-C12-C13	120.4(7)
O3-C5-C4	117.69(17)	C11X-C12X-C13X	123.5(10)
C4-C5-C6	122.19(17)	C12-C13-C14	119.5(7)
O3-C5-C6	120.00(16)	C12X-C13X-C14X	119.6(10)
C1-C6-C5	116.81(16)	C13-C14-C15	120.7(7)
C5-C6-C7	124.51(16)	C13X-C14X-C15X	120.1(10)
C1-C6-C7	118.62(17)	C10-C15-C14	119.2(6)
O4-C7-C8	119.5(2)	C10X-C15X-C14X	120.5(9)
O4-C7-C8X	118.6(3)	C7-C8-H8A	110.00
C6-C7-C8	114.67(19)	C7-C8-H8B	110.00
C6-C7-C8X	115.2(3)	C9-C8-H8A	110.00
O4-C7-C6	125.26(19)	C9-C8-H8B	110.00
C7-C8-C9	108.7(3)	H8A-C8-H8B	108.00
C7-C8X-H8XA	109.00	C13X-C14X-H14B	120.00
C7-C8X-H8XB	109.00	C15X-C14X-H14B	120.00
C9X-C8X-H8XA	109.00	C14-C15-H15A	120.00
C9X-C8X-H8XB	109.00	C10-C15-H15A	120.00
H8XA-C8X-H8XB	108.00	C14X-C15X-H15B	120.00
C10-C9-H9A	110.00	C10X-C15X-H15B	120.00
O1 -C9-H9A	110.00	C2-C16-H16B	109.00
C8-C9-H9A	110.00	C2-C16-H16A	109.00
O1-C9X-H9XA	108.00	H16B-C16-H16C	109.00
C8X-C9X-H9XA	108.00	H16A-C16-H16C	109.00
C10X-C9X-H9XA	108.00	C2-C16-H16C	109.00
C10-C11-H11A	118.00	H16A-C16-H16B	109.00
C12-C11-H11A	118.00	C4-C17-H17B	109.00
C10X-C11X-H11B	121.00	H17A-C17-H17C	109.00
C12X-C11X-H11B	121.00	C4-C17-H17C	109.00

Table S6. *Cont.*

Bond Angles (Degrees)			
C11-C12-H12A	120.00	H17A-C17-H17B	109.00
C13-C12-H12A	120.00	C4-C17-H17A	109.00
C11X-C12X-H12B	118.00	H17B-C17-H17C	110.00
C13X-C12X-H12B	118.00	O3-C18-H18C	110.00
C12-C13-H13A	120.00	H18A-C18-H18C	109.00
C14-C13-H13A	120.00	H18B-C18-H18C	109.00
C12X-C13X-H13B	120.00	H18A-C18-H18B	109.00
C14X-C13X-H13B	120.00	O3-C18-H18A	109.00
C13-C14-H14A	120.00	O3-C18-H18B	109.00
C15-C14-H14A	120.00		

Table S7. Torsion Angles (Degrees) for: mo_f16_0m P 21/c, R = 0.08.

Torsion Angles (Degrees)	
C9-O1-C1-C2	−155.07(19)
C9-O1-C1-C6	25.3(3)
C1-O1-C9-C8	−58.3(3)
C1-O1-C9-C10	180.0(3)
C18-O3-C5-C4	101.9(2)
C18-O3-C5-C6	−82.1(2)
O1-C1-C2-C3	179.24(13)
O1-C1-C2-C16	−1.3(2)
C6-C1-C2-C3	−1.2(2)
C6-C1-C2-C16	178.33(17)
O1-C1-C6-C5	−179.29(16)
O1-C1-C6-C7	3.5(3)
C2-C1-C6-C5	1.2(3)
C2-C1-C6-C7	−176.06(16)
C1-C2-C3-O2	−179.49(14)
C1-C2-C3-C4	−0.2(2)
C16-C2-C3-O2	1.0(2)
C16-C2-C3-C4	−179.68(17)
O2-C3-C4-C5	−179.18(17)
O2-C3-C4-C17	1.4(3)
C2-C3-C4-C5	1.5(3)
C2-C3-C4-C17	−177.95(18)
C3-C4-C5-O3	174.45(17)
C3-C4-C5-C6	−1.5(3)
C17-C4-C5-O3	−6.1(3)
C17-C4-C5-C6	178.0(2)
O3-C5-C6-C1	−175.62(17)
O3-C5-C6-C7	1.4(3)
C4-C5-C6-C1	0.2(3)
C4-C5-C6-C7	177.3(2)
C1-C6-C7-C8	5.1(3)
C1-C6-C7-O4	176.6(2)
C5-C6-C7-O4	−0.4(4)

Table S7. *Cont.*

Torsion Angles (Degrees)	
C5-C6-C7-C8	−171.9(2)
O4-C7-C8-C9	19.6(2)
C6-C7-C8-C9	−38.4(3)
C7-C8-C9-O1	63.5(3)
C7-C8-C9-C10	−177.8(3)
O1-C9-C10-C11	−153.0(6)
O1-C9-C10-C15	32.3(6)
C8-C9-C10-C11	89.9(7)
C8-C9-C10-C15	−84.7(6)
C5-C6-C7-O4	−0.4(4)
C5-C6-C7-C8	−171.9(2)
O4-C7-C8-C9	149.6(2)
C6-C7-C8-C9	−38.4(3)
C7-C8-C9-O1	63.5(3)
C7-C8-C9-C10	−177.8(3)
O1-C9-C10-C11	−153.0(6)
O1-C9-C10-C15	32.3(6)
C8-C9-C10-C11	89.9(7)
C8-C9-C10-C15	−84.7(6)
C9-C10-C11-C12	178.7(6)

Table S8. Contact distances (angstrom) for: mo_f16_0m P 21/c, R = 0.08.

Contact Distances (Angstrom)			
O2.C8X_b	3.382(7)	C8X.O2_e	3.382(7)
O2.C9_c	3.402(4)	C9.O2_c	3.402(4)
O2.O4_b	2.820(2)	C14.C14_h	3.322(10)
O3.O4	2.763(2)	C14.C15_h	3.309(10)
O4.O2_e	2.820(2)	C15.C14_h	3.309(10)
O4.C18	2.977(3)	C16.O4_b	3.258(3)
O4.O3	2.763(2)	C16.C5_a	3.580(3)
O4.C16_e	3.258(3)	C17.C18	3.527(3)
O1.H17C_a	2.9000	C18.C17	3.527(3)
O1.H16A	2.2700	C18.C7	3.380(3)
O1.H15A	2.5400	C18.O4	2.977(3)
O2.H8B_b	2.8000	C1.H8XB	3.0800
O2.H17B	2.5000	C1.H8A	3.0300
O2.H9A_c	2.5200	C5.H16C_c	3.0200
O2.H8XA_b	2.8000	C5.H16B_a	2.8000
O2.H16C	2.7200	C6.H18B	2.8900
O3.H16B_a	2.5700	C6.H9A	2.8900
O3.H13B_d	2.8000	C6.H9XA	3.0800
O3.H17A	2.4100	C7.H18B	2.8700
O4.H18B	2.5000	C7.H1O2_e	2.88(3)
O4.H1O2_e	2.03(3)	C8.H11A_f	2.9300
O4.H16C_e	2.5900	C8X.H1O2_e	3.09(3)
O4.H18B_f	2.8000	C8X.H8XB_f	3.0600
O4.H8XB_f	2.9100	C9X.H17C_a	3.0500

Table S8. *Cont.*

Contact Distances (Angstrom)			
C1.C3_a	3.595(2)	C11X.H8XB	3.0200
C3.C1_a	3.595(2)	C11X.H17B_c	2.9300
C5.C16_a	3.580(3)	C13.H18A_g	3.0100
C7.C18	3.380(3)	C13X.H18A_g	3.0700
C14.H14A_h	3.0400	H8B.H11A_f	2.4500
C14.H15A_h	3.0600	H8B.H9A_f	2.5800
C15.H14A_h	2.8100	H8B.O2_e	2.8000
C15.H17C_a	3.1000	H9A.O2_c	2.5200
C16.H1O2	2.40(4)	H9A.H1O2_c	2.6000
C17.H15A_a	3.0900	H9A.H11A	2.4400
C18.H17A	2.8800	H9A.C6	2.8900
C18.H16C_c	3.0300	H9A.H8B_i	2.5800
H8XA.H11B_f	2.5300	H11A.H9A	2.4400
H8XA.H8XB_f	2.3700	H11A.C8_i	2.9300
H8XA.O2_e	2.8000	H11A.H8A_i	2.6000
H8XB.O4_i	2.9100	H11A.H8B_i	2.4500
H8XB.C1	3.0800	H11B.H17B_c	2.4600
H8XB.C11X	3.0200	H11B.H8XA_i	2.5300
H8XB.C8X_i	3.0600	H13A.H18A_g	2.4500
H8XB.H8XA_i	2.3700	H13B.O3_k	2.8000
H9XA.H15B	1.8900	H13B.H18A_g	2.5500
H9XA.C6	3.0800	H14A.C14_h	3.0400
H9XA.H17C_a	2.4200	H14A.C15_h	2.8100
H1O2.C16	2.40(4)	H15A.O1	2.5400
H1O2.H16B	2.4700	H15A.C14_h	3.0600
H1O2.O4_b	2.03(3)	H15A.H17C_a	2.1800
H1O2.C7_b	2.88(3)	H15A.C17_a	3.0900
H1O2.C8X_b	3.09(3)	H15B.H9XA	1.8900
H1O2.H9A_c	2.6000	H16A.O1	2.2700
H1O2.H16C	2.0600	H16B.C5_a	2.8000
H8A.C1	3.0300	H16B.H1O2	2.4700
H8A.H11A_f	2.6000	H16B.O3_a	2.5700
H16C.H1O2	2.0600	H17C.C15_a	3.1000
H16C.O2	2.7200	H17C.H15A_a	2.1800
H16C.O4_b	2.5900	H17C.C9X_a	3.0500
H16C.C5_c	3.0200	H18A.H13A_j	2.4500
H16C.C18_c	3.0300	H18A.C13X_j	3.0700
H17A.O3	2.4100	H18A.H13B_j	2.5500
H17A.C18	2.8800	H18A.C13_j	3.0100
H17A.H18C	2.4400	H18B.O4	2.5000
H17B.O2	2.5000	H18B.C6	2.8900
H17B.O2	2.5000	H18B.C6	2.8900
H17B.O2	2.5000	H18B.C6	2.8900
H17B.C11X_c	2.9300	H18B.O4_i	2.8000
H17B.H11B_c	2.4600	H18B.C7	2.8700
H17C.H9XA_a	2.4200	H18C.H17A	2.4400
H17C.O1_a	2.9000		

Table S9. Hydrogen bonds (angstrom, Deg) for: mo_f16_0m P 21/c, R = 0.08.

Hydrogen Bonds (Angstrom, Deg)				
O2--H1O2...O4	0.88(4)	2.03(3)	2.820(2)	150(3) 2_655
C9--H9A...O2	0.9800	2.5200	3.402(4)	150.00 3_666
C16--H16A...O1	0.9600	2.2700	2.721(2)	108.00
C16--H16B...O3	0.9600	2.5700	3.504(3)	163.00 3_665
C16--H16C...O4	0.9600	2.5900	3.258(3)	127.00 2_655
C17--H17A...O3	0.9600	2.4100	2.807(3)	104.00
C18--H18B...O4	0.9600	2.5000	2.977(3)	110.00

Translation of Symmetry Code to Equiv. Pos. a = [3665.00] = 1 − x, 1 − y, −z; b = [2655.00] = 1 − x, /2 + y, 1/2 − z; c = [3666.00] = 1 − x, 1 − y, 1 − z; d = [1655.00] = 1 + x, y, z; e = [2645.00] = 1 − x, −1/2 + y, 1/2 − z; f = [4554.00] = x, 1/2 − y, −1/2 + z; g = [4454.00] = −1 + x, 1/2 − y, −1/2 + z; h = [3565.00] = −x, 1 − y, −z; i= [4555.00] = x, 1/2 − y, 1/2 + z; j = [4655.00] = 1 + x, 1/2 − y, 1/2 + z; k = [1455.00] = −1 + x, y ,z.

Supplementary material of compound-2: (*S*)-5,7-dihydroxy-6,8-dimethyl flavanone.

Table S10. Crystal data and details of the structure determination for: cu_rf4_0m P 21/c, R = 0.06.

Table S11. Final coordinates and equivalent isotropic displacement parameters of the non-hydrogen atoms for: cu_rf4_0m P 21/c, R = 0.06.

Table S12. Hydrogen atom positions and isotropic displacement parameters for: cu_rf4_0m P 21/c, R = 0.06.

Table S13. (An) isotropic displacement parameters for: cu_rf4_0m P 21/c, R = 0.06.

Table S14. Bond distances (angstrom) for: cu_rf4_0m P 21/c, R = 0.06.

Table S15. Bond angles (degrees) for: cu_rf4_0m P 21/c, R = 0.06.

Table S16. Torsion angles (degrees) for: cu_rf4_0m P 21/c, R = 0.06.

Table S17. Contact distances (angstrom) for: cu_rf4_0m P 21/c, R = 0.06.

Table S18. Hydrogen bonds (angstrom, Deg) for: cu_rf4_0m P 21/c, R = 0.06.

Table S10. Crystal Data and Details of the Structure Determination for: cu_rf4_0m P 21/c, R = 0.06.

Crystal Data	
Formula	C17H16O4
Formula Weight	284.30
Crystal System	Monoclinic
Space group	P21/c (No. 14)
a, b, c [Angstrom]	4.8133 (1), 24.5685 (6), 12.7303 (4)
alpha, beta, gamma [deg]	90, 100.1616 (17), 90
V [Å^3]	1481.82 (7)
Z	4
D(calc) [g/cm^3]	1.274
Mu(CuKa) [/mm]	0.744
F(000)	600
Crystal Size [mm]	0.03 × 0.09× 0.32
Data Collection	
Temperature (K)	294
Radiation [Angstrom]	CuKa 1.54178
Theta Min-Max [Deg]	4.0, 70.2
Dataset	−5:5; −28:29; −15:14
Tot., Uniq. Data, R(int)	26315, 2751, 0.037
Observed data [I > 2.0 sigma (I)]	2104
Refinement	
Nref, Npar	2751, 240
R, wR2, S	0.0562, 0.1714, 1.10
w = ^2^(FO^2^) + (0.0838P)^2^ + 0.3993P]	WHERE P = (FO^2^ + 2FC^2^)/3′
Max. and Av. Shift/Error	0.00, 0.00
Min. and Max. Resd. Dens. [e/Ang^3]	−0.21, 0.24

Table S11. Final Coordinates and Equivalent Isotropic Displacement Parameters of the non-Hydrogen atoms for: cu_rf4_0m P 21/c, R = 0.06

Atom	X	Y	Z	U (eq) [Ang^2]
O1	0.1456(3)	0.64746(5)	0.59881(10)	0.0537(5)
O2	0.4666(4)	0.79909(7)	0.42296(13)	0.0680(6)
O3	−0.0674(4)	0.83136(7)	0.68834(13)	0.0695(6)
O4	−0.2424(3)	0.74901(6)	0.78283(12)	0.0691(6)
C1	0.1622(4)	0.70269(8)	0.58604(14)	0.0451(6)
C2	0.3063(4)	0.72074(8)	0.50797(14)	0.0470(6)
C3	0.3268(4)	0.77743(9)	0.49614(15)	0.0513(6)
C4	0.2080(4)	0.81530(8)	0.55692(16)	0.0558(7)
C5	0.0568(4)	0.79524(8)	0.63155(16)	0.0528(7)
C6	0.0330(4)	0.73891(8)	0.64884(15)	0.0484(6)
C7	−0.1220(4)	0.71838(9)	0.72700(16)	0.0541(7)
C8	−0.1377(5)	0.65840(10)	0.7369(2)	0.0714(9)
* C9	0.1108(12)	0.6294(2)	0.7043(4)	0.0474(16)
* C10	0.0577(13)	0.56607(17)	0.6894(4)	0.072(3)
* C11	0.1805(17)	0.5340(3)	0.7751(3)	0.102(3)
* C12	0.1477(18)	0.4778(2)	0.7705(4)	0.111(3)
* C13	−0.0080(14)	0.45365(16)	0.6802(6)	0.118(4)
* C14	−0.1308(14)	0.48568(19)	0.5945(6)	0.116(3)
* C15	−0.0980(14)	0.54189(18)	0.5991(5)	0.092(2)
C16	0.4331(5)	0.68193(9)	0.43819(17)	0.0592(7)
C17	0.2394(6)	0.87549(10)	0.5423(2)	0.0802(10)
* C15X	−0.2069(17)	0.5400(3)	0.6416(6)	0.103(3)
* C9X	−0.035(3)	0.6273(3)	0.6678(10)	0.094(4)
* C10X	0.0232(18)	0.5706(2)	0.6901(6)	0.085(3)
* C11X	0.2599(15)	0.5449(3)	0.7469(8)	0.118(4)
* C12X	0.2666(17)	0.4884(3)	0.7553(8)	0.143(4)
* C13X	0.0365(19)	0.4578(2)	0.7069(7)	0.130(5)
* C14X	−0.2003(16)	0.4836(3)	0.6500(8)	0.131(4)

U (eq) = 1/3 of the trace of the orthogonalized U Tensor Starred Atom sites have a S.O.F less than 1.0.
* Note: Carbon atom showed statistical conformational disorders.

Table S12. Hydrogen Atom Positions and Isotropic Displacement Parameters for: cu_rf4_0m P 21/c, R = 0.06

Atom	X	Y	Z	U (iso) [Ang^2]
H1O2	0.547(7)	0.7751(13)	0.388(3)	0.106(10)
* H14A	−0.23490	0.46950	0.53410	0.1390
* H15A	−0.18010	0.56330	0.54170	0.1100
H16A	0.39430	0.64520	0.45680	0.0890
H16B	0.63350	0.68740	0.44840	0.0890
H16C	0.35230	0.68840	0.36480	0.0890
* H17A	0.17230	0.89460	0.59870	0.1210
* H17B	0.13120	0.88630	0.47490	0.1210
* H17C	0.43470	0.88410	0.54400	0.1210
H1O3	−0.164(6)	0.8110(14)	0.731(3)	0.109(11)
* H8A	−0.31020	0.64560	0.69270	0.0860
* H8B	−0.14630	0.64920	0.81040	0.0860
* H9A	0.28310	0.63630	0.75620	0.0570
* H11A	0.28460	0.55020	0.83560	0.1230
* H12A	0.22990	0.45640	0.82790	0.1330
* H13A	−0.02990	0.41600	0.67710	0.1420
* H9XA	−0.20770	0.62310	0.61480	0.1120
* H8C	−0.04100	0.64860	0.80780	0.0860
* H8D	−0.33480	0.64880	0.73290	0.0860
* H11B	0.41390	0.56530	0.77930	0.1410
* H12B	0.42500	0.47120	0.79330	0.1710
* H13B	0.04090	0.42010	0.71250	0.1560
* H14B	−0.35420	0.46310	0.61760	0.1570
* H15B	−0.36530	0.55730	0.60360	0.1240
* H17D	0.32320	0.89160	0.60910	0.1210
* H17E	0.05700	0.89140	0.51820	0.1210
* H17F	0.35800	0.88200	0.49040	0.1210

The Temperature Factor has the Form of Exp (−T) Where T = 8 * (Pi ** 2) * U * (Sin (Theta)/Lambda) ** 2 for Isotropic Atoms. * Note: Hydrogen atom showed statistical conformational disorders.

Table S13. (An) Isotropic Displacement Parameters for: cu_rf4_0m P 21/c, R = 0.06.

Atom	U(1,1) or U(2,2)	U(3,3)	U(2,3)	U(1,3)	U(1,2)
O1	0.0651(9) 0.0473(8)	0.0558(8)	0.0018(6)	0.0304(7)	−0.0012(6)
O2	0.0874(11) 0.0597(10)	0.0687(10)	0.0067(8)	0.0465(9)	−0.0034(8)
O3	0.0897(12) 0.0570(9)	0.0715(10)	−0.0083(7)	0.0412(9)	0.0066(8)
O4	0.0804(11) 0.0739(11)	0.0649(9)	−0.0040(7)	0.0453(8)	0.0053(8)
C1	0.0474(11) 0.0460(11)	0.0447(10)	0.0004(8)	0.0159(8)	−0.0009(7)
C2	0.0492(11) 0.0523(11)	0.0434(10)	−0.0007(8)	0.0187(8)	−0.0006(8)
C3	0.0555(11) 0.0554(12)	0.0474(10)	0.0033(9)	0.0214(8)	−0.0035(9)
C4	0.0665(13) 0.0504(12)	0.0545(12)	0.0005(9)	0.0214(10)	−0.0012(9)
C5	0.0599(12) 0.0531(12)	0.0496(11)	−0.0060(9)	0.0214(9)	0.0021(9)
C6	0.0515(11) 0.0525(11)	0.0461(10)	−0.0014(8)	0.0217(8)	0.0006(8)
C7	0.0531(12) 0.0629(13)	0.0518(11)	−0.0016(9)	0.0244(9)	0.0019(9)
C8	0.0851(17) 0.0656(15)	0.0763(15)	0.0082(12)	0.0493(13)	−0.0004(11)
C9	0.037(3) 0.057(2)	0.051(3)	0.0079(18)	0.0155(19)	0.0015(19)
C10	0.101(5) 0.045(3)	0.083(5)	0.014(3)	0.054(4)	0.009(3)
C11	0.148(6) 0.073(4)	0.089(4)	0.020(3)	0.029(4)	−0.016(4)
C12	0.140(7) 0.073(4)	0.122(5)	0.032(4)	0.029(5)	−0.008(4)
C13	0.141(7) 0.068(5)	0.147(7)	0.011(4)	0.028(6)	−0.003(5)
C14	0.153(6) 0.080(4)	0.116(5)	−0.002(4)	0.029(4)	−0.016(4)
C15	0.106(5) 0.069(3)	0.103(4)	0.008(3)	0.028(3)	−0.003(3)
C16	0.0698(13) 0.0597(13)	0.0557(12)	−0.0031(10)	0.0323(10)	0.0007(10)
C17	0.114(2) 0.0514(14)	0.0853(18)	−0.0006(12)	0.0454(15)	−0.0049(13)
C15X	0.122(6) 0.095(4)	0.102(5)	0.005(4)	0.044(4)	0.008(4)
C9X	0.130(8) 0.061(3)	0.113(7)	0.013(4)	0.086(6)	0.003(4)
C10X	0.114(6) 0.064(5)	0.096(7)	0.009(4)	0.070(5)	0.008(4)
C11X	0.129(6) 0.074(4)	0.160(8)	0.044(5)	0.050(5)	−0.006(4)
C12X	0.151(7) 0.082(6)	0.206(9)	0.069(6)	0.063(7)	0.028(5)
C13X	0.227(10) 0.045(5)	0.139(8)	0.018(5)	0.088(8)	−0.006(5)
C14X	0.185(8) 0.098(5)	0.118(7)	−0.017(6)	0.052(6)	−0.034(5)
C14X	0.185(8) 0.098(5)	0.118(7)	−0.017(6)	0.052(6)	−0.034(5)

The Temperature Factor has the Form of Exp (−T) Where T = 8 * (Pi ** 2) * U * (Sin(Theta)/Lambda) ** 2 for Isotropic Atoms T = 2 * (Pi ** 2) * Sumij(h(i) * h(j) * U(i,j) * Astar(i) * Astar(j)), for An isotropic Atoms. Astar (i) are Reciprocal Axial Lengths and h (i) are the Reflection Indices.

Table S14. Bond Distances (Angstrom) for: cu_rf4_0m P 21/c, R = 0.06.

**Bond Distances (Angstrom)**			
O1-C1	1.371(2)	C12-C13	1.390(9)
O1-C9	1.452(5)	C12X-C13X	1.390(12)
O1-C9X	1.429(13)	C13-C14	1.390(9)
O2-C3	1.351(3)	C13X-C14X	1.391(12)
O3-C5	1.349(3)	C14-C15	1.390(6)
O4-C7	1.246(3)	C14X-C15X	1.390(10)
O2-H1O2	0.87(3)	C8-H8A	0.9700
O3-H1O3	0.92(3)	C8-H8B	0.9700
C1-C2	1.382(3)	C8-H8C	0.9700
C1-C6	1.412(3)	C8-H8D	0.9700
C2-C16	1.504(3)	C9-H9A	0.9800
C2-C3	1.406(3)	C9X-H9XA	0.9800
C3-C4	1.396(3)	C11-H11A	0.9300
C4-C17	1.501(3)	C11X-H11B	0.9300
C4-C5	1.385(3)	C12-H12A	0.9300
C5-C6	1.409(3)	C12X-H12B	0.9300
C6-C7	1.436(3)	C13-H13A	0.9300
C7-C8	1.482(3)	C13X-H13B	0.9300
C8-C9	1.512(6)	C14-H14A	0.9300
C8-C9X	1.325(12)	C14X-H14B	0.9300
C9-C10	1.583(7)	C15-H15A	0.9300
C9X-C10X	1.439(9)	C15X-H15B	0.9300
C10-C15	1.390(8)	C16-H16B	0.9600
C10-C11	1.390(7)	C16-H16A	0.9600
C10X-C15X	1.390(11)	C16-H16C	0.9600
C10X-C11X	1.389(11)	C17-H17F	0.9600
C11-C12	1.390(9)	C17-H17A	0.9600
C11X-C12X	1.392(10)	C17-H17B	0.9600
C17-H17C	0.9600	C17-H17E	0.9600
C17-H17D	0.9600		

Table S15. Bond angles (degrees) for: cu_rf4_0m P 21/c, R = 0.06.

Bond Angles (Degrees)			
C1-O1-C9	115.5(2)	O1-C9-C8	109.6(3)
C1-O1-C9X	118.1(4)	O1-C9-C10	103.4(4)
C3-O2-H1O2	114(2)	C8-C9X-C10X	120.6(9)
C5-O3-H1O3	106(2)	O1-C9X-C8	123.2(6)
C2-C1-C6	122.16(18)	O1-C9X-C10X	109.8(9)
O1-C1-C6	121.07(17)	C9-C10-C11	115.2(5)
O1-C1-C2	116.76(17)	C11-C10-C15	120.0(5)
C1-C2-C3	116.65(17)	C9-C10-C15	124.9(4)
C1-C2-C16	121.93(18)	C11X-C10X-C15X	120.1(6)
C3-C2-C16	121.41(17)	C9X-C10X-C11X	131.0(8)
O2-C3-C2	121.12(18)	C9X-C10X-C15X	108.9(8)
C2-C3-C4	123.89(18)	C10-C11-C12	120.0(5)
O2-C3-C4	114.99(19)	C10X-C11X-C12X	119.9(7)
C3-C4-C17	121.86(19)	C11-C12-C13	120.0(5)
C3-C4-C5	117.34(18)	C11X-C12X-C13X	120.0(8)
C5-C4-C17	120.80(19)	C12-C13-C14	120.0(4)
O3-C5-C6	120.42(18)	C12X-C13X-C14X	120.0(6)
O3-C5-C4	118.01(18)	C13-C14-C15	120.0(6)
C4-C5-C6	121.57(18)	C13X-C14X-C15X	120.0(7)
C5-C6-C7	121.30(18)	C10-C15-C14	120.0(5)
C1-C6-C5	118.33(17)	C10X-C15X-C14X	120.0(7)
C1 -C6-C7	120.36(18)	C7-C8-H8A	109.00
O4-C7-C8	121.08(19)	C7-C8-H8B	109.00
O4-C7-C6	122.3(2)	C7-C8-H8C	108.00
C6-C7-C8	116.65(19)	C7-C8-H8D	108.00
C7-C8-C9X	119.1(5)	C9-C8-H8A	109.00
C7-C8-C9	113.0(3)	C9-C8-H8B	109.00
C8-C9-C10	112.2(4)	H8A-C8-H8B	108.00
C9X-C8-H8C	107.00	C15X-C14X-H14B	120.00
C9X-C8-H8D	108.00	C14-C15-H15A	120.00
H8C-C8-H8D	107.00	C10-C15-H15A	120.00
O1-C9-H9A	110.00	C10X-C15X-H15B	120.00
C8-C9-H9A	110.00	C14X-C15X-H15B	120.00
C10-C9-H9A	111.00	C2-C16-H16C	109.00
C10X-C9X-H9XA	98.00	C2-C16-H16B	110.00
C8-C9X-H9XA	98.00	C2-C16-H16A	109.00
O1-C9X-H9XA	98.00	H16A-C16-H16C	109.00
C10-C11-H11A	120.00	H16B-C16-H16C	109.00
C12 -C11-H11A	120.00	H16A-C16-H16B	109.00
C12X-C11X-H11B	120.00	H17D-C17-H17F	109.00
C10X-C11X -H11B	120.00	H17E-C17-H17F	109.00
C11-C12-H12A	120.00	C4-C17-H17A	110.00
C13-C12-H12A	120.00	C4-C17-H17B	109.00
C11X-C12X-H12B	120.00	C4-C17-H17C	109.00

Table S15. *Cont.*

Bond Angles (Degrees)			
C13X-C12X-H12B	120.00	C4-C17-H17D	109.00
C14-C13-H13A	120.00	C4-C17-H17E	110.00
C12-C13-H13A	120.00	C4-C17-H17F	110.00
C14X-C13X-H13B	120.00	H17A-C17-H17B	109.00
C12X-C13X-H13B	120.00	H17A-C17-H17C	109.00
C13-C14-H14A	120.00	H17B-C17-H17C	110.00
C15-C14-H14A	120.00	H17D-C17-H17E	109.00
C13X-C14X-H14B	120.00		

Table S16. Torsion Angles (Degrees) for: cu_rf4_0m P 21/c, R = 0.06.

Torsion Angles (Degrees)	
C9-O1-C1-C2	154.0(3)
C9-O1-C1-C6	−27.1(3)
C1-O1-C9-C8	52.9(4)
C1-O1-C9-C10	172.7(3)
O1-C1-C2-C3	−179.05(16)
O1-C1-C2-C16	1.5(3)
C6-C1-C2-C3	2.1(3)
C6-C1-C2-C16	−177.39(19)
O1-C1-C6-C5	−179.75(17)
O1-C1-C6-C7	−0.7(3)
C2-C1-C6-C5	−0.9(3)
C2-C1-C6-C7	178.13(18)
C1-C2-C3-O2	179.14(18)
C1-C2-C3-C4	−1.0(3)
C16-C2-C3-O2	−1.4(3)
C16-C2-C3-C4	178.51(19)
O2-C3-C4-C5	178.59(18)
O2-C3-C4-C17	−1.0(3)
C2-C3-C4-C5	−1.3(3)
C2-C3-C4-C17	179.1(2)
C3-C4-C5-O3	−177.77(18)
C3-C4-C5-C6	2.6(3)
C17-C4-C5-O3	1.8(3)
C17-C4-C5-C6	−177.9(2)
O3-C5-C6-C1	178.83(18)
O3-C5-C6-C7	−0.2(3)
C4-C5-C6-C1	−1.5(3)
C4-C5-C6-C7	179.45(19)
C1-C6-C7-O4	−178.92(18)
C5-C6-C7-O4	0.1(3)
C1-C6-C7-C8	−0.2(3)
C5-C6-C7-C8	178.85(19)
O4-C7-C8-C9	−154.2(3)
C6-C7-C8 -C9	27.1(3)
C7-C8-C9-O1	−52.4(4)
C7-C8-C9-C10	−166.6(3)

Table S16. *Cont.*

Torsion Angles (Degrees)	
O1-C9-C10-C11	142.5(5)
O1-C9-C10-C15	−37.5(7)
C8-C9-C10-C11	−99.5(6)
C8-C9-C10-C15	80.6(7)
C9-C10-C11-C12	−179.9(6)
C15-C10-C11-C12	0.0(11)
C9-C10-C15-C14	179.9(6)
C11-C10-C15-C14	0.0(10)
C10-C11-C12-C13	0.0(12)
C11-C12-C13-C14	0.0(11)
C12-C13-C14-C15	0.0(11)
C13-C14-C15-C10	0.0(10)

Table S17. Contact distances (angstrom) for: cu_rf4_0m P 21/c, R = 0.06.

Contact Distances (Angstrom)			
O2.O4_b	2.724(2)	C2.C7_a	3.556(3)
O3.C13X_d	3.374(6)	C7.C2_g	3.556(3)
O3.O4	2.571(2)	C11X.C14X_a	3.416(11)
O4.O2_f	2.724(2)	C11X.C15X_a	3.100(11)
O4.C16_f	3.213(3)	C12X.C15X_a	3.379(12)
O4.O3	2.571(2)	C12X.C14X_a	3.100(12)
O1.H15A	2.6200	C13X.O3_i	3.374(6)
O1.H8A_a	2.6800	C14.C15_h	2.950(10)
O1.H16A	2.3400	C14.C14_h	2.990(10)
O1.H8D_a	2.7700	C14X.C12X_g	3.100(12)
O2.H16C	2.8500	C14X.C11X_g	3.416(11)
O2.H16B	2.8600	C15.C14_h	2.950(10)
O2.H8C_c	2.9200	C15.C15_h	3.512(8)
O2.H17C	2.6200	C15X.C12X_g	3.379(12)
O2.H17B	2.8300	C15X.C11X_g	3.100(11)
O2.H9A_c	2.6700	C16.O4_b	3.213(3)
O2.H17F	2.3100	C1.H16B_g	2.8500
O2.H8B_b	2.8400	C1.H8D_a	3.0800
O3.H17D	2.7200	C1.H8A_a	3.0100
O3.H13B_d	2.5100	C5.H16C_e	3.0800
O3.H16C_e	2.7900	C6.H9XA	3.0700
O3.H17A	2.3500	C6.H9A	3.0200
O3.H17E	2.7700	C7.H1O2_f	2.81(4)
O3.H13A_d	2.6800	C7.H1O3	2.29(3)
O4.H1O3	1.73(3)	C8.H9A_g	2.8900
O4.H1O2_f	1.91(4)	C8.H15B	3.1000
O4.H16B_f	2.7700	C9.H8A_a	2.8400
O4.H16C_f	2.8200	C11X.H15B_a	2.8000
C11X.H8C	3.1000	H8B.H17B_e	2.4400
C12X.H14B_a	2.8100	H8C.C11X	3.1000
C14.H15A_h	2.7600	H8C.O2_e	2.9200
C14.H14A_h	2.8300	H8D.C1_g	3.0800
C14X.H12B_g	2.8000	H8D.C15X	3.0200

Table S17. *Cont.*

Contact Distances (Angstrom)			
C15.H8A	3.0600	H8D.O1_g	2.7700
C15.H14A_h	2.5500	H8D.H11B_g	2.5000
C15X.H11B_g	2.8100	H9A.C8_a	2.8900
C15X.H8D	3.0200	H9A.H8A_a	2.2600
C16.H1O2	2.46(3)	H9A.C6	3.0200
C16.H9XA_a	2.9600	H9A.O2_e	2.6700
H1O2.C16	2.46(3)	H9A.H11A	2.3400
H1O2.H16B	2.3000	H11A.H9A	2.3400
H1O2.H16C	2.3200	H11A.H17B_e	2.5700
H1O2.O4_b	1.91(4)	H11B.H8D_a	2.5000
H16B.O2	2.8600	H17C.O2	2.6200
H1O2.C7_b	2.81(4)	H11B.C15X_a	28100
H1O3.O4	1.73(3)	H12B.C14X_a	2.8000
H1O3.C7	2.29(3)	H12B.H17D_j	2.5100
H9XA.C16_g	2.9600	H13A.O3_i	2.6800
H9XA.H16A_g	2.5800	H13B.O3_i	2.5100
H9XA.H15B	1.7800	H14A.C14_h	2.8300
H9XA .C6	3.0700	H14A.C15_h	2.5500
H8A.H9A_g	2.2600	H14A.H15A_h	2.5000
H8A.C1_g	3.0100	H14B.C12X_g	2.8100
H8A.C15	3.0600	H15A.O1	2.6200
H8A.O1_g	2.6800	H15A.C14_h	2.7600
H8A.C9_g	2.8400	H15A.H14A_h	2.5000
H8B.O2_f	2.8400	H15B.H9XA	1.7800
H15B.C8	3.1000	H16C.O4_b	2.8200
H15B.C11X_g	2.8000	H16C.C5_c	3.0800
H16A.O1	2.3400	H17A.O3	2.3500
H16A.H9XA_a	2.5800	H17B.O2	2.8300
H16B.H1O2	2.3000	H17B.H8B_c	2.4400
H16B.O4_b	2.7700	H17B.H11A_c	2.5700
H16B.C1_a	2.8500	H17D.O3	2.7200
16C.O2	2.8500	H17D.H12B_k	2.5100
H16C.H1O2	2.3200	H17E.O3	2.7700
H16C.O3_c	2.7900	H17F.O2	2.3100

Table S18. Hydrogen Bonds (Angstrom, Deg) for: cu_rf4_0m P 21/c, R = 0.06.

Hydrogen Bonds (Angstrom, Deg)					
O2.....H1O2...O4	0.87(3)	1.91(4)	2.724(2)	155(3)	4_664
O3......H1O3...O4	0.92(3)	1.73(3)	2.571(2)	150(3)	
C16.....H16A...O1	0.9600	2.3400	2.795(3)	108.00	
C17....H17A...O3	0.9600	2.3500	2.790(3)	107.00	

Translation of Symmetry Code to Equiv. Pos. a = [1655.00] = 1 + x, y, z; b = [4664.00] = 1 + x, 3/2 − y, −1/2 + z; c = [4564.00] = x, 3/2 − y, −1/2 + z; d = [2556.00] = −x, ½ + y, 3/2 − z; e = [4565.00] = x, 3/2 − y, 1/2 + z; f = [4465.00] = −1 + x, 3/2 − y, 1/2 + z; g = [1455.00] = −1 + x, y, z; h = [3566.00] = −x, 1 − y, 1 − z; i = [2546.00] = −x, −1/2 + y, 3/2 − z; j = [2646.00] = 1 − x, −1/2 + y, 3/2 − z; k = [2656.00] = 1 − x, 1/2 + y, 3/2 − z.

Table S19. Robustness results of compounds (**1**–**5**).

Compound	Parameters	Optimized	Used	Retention Time (min)	Peak Asymmetry	Remarks
1	a. Flow rate (±0.1 mL·min^{-1})	1.0 (mL·min^{-1})	0.9 (mL·min^{-1})<br>1.1 (mL·min^{-1})	5.6<br>4.6	0.84<br>0.88	Robust
	b. Detection wavelength (± 5 nm)	210 (nm)	205 (nm)<br>215 (nm)	5.2<br>5.2	0.84<br>0.85	Robust
	c. Mobile Phase composition: Acetonitrile contents (±5%)	60:40 *v*/*v*	55:45 *v*/*v*<br>65:35 *v*/*v*	6.2<br>4.2	0.84<br>0.84	Robust
	d. Column Temperature (±5 °C)	30 °C	25 °C<br>35 °C	5.2<br>5.2	0.85<br>0.86	Robust
2	a. Flow rate (±0.1 mL·min^{-1})	1.0 (mL·min^{-1})	0.9 (mL·min^{-1})<br>1.1 (mL·min^{-1})	8.9<br>6.6	0.90<br>0.94	Robust
	b. Detection wavelength (±5 nm)	210 (nm)	205 (nm)<br>215 (nm)	7.4<br>7.3	0.90<br>0.93	Robust
	c. Mobile Phase composition: Acetonitrile contents (±5%)	60:40 *v*/*v*	55:45 *v*/*v*<br>65:35 *v*/*v*	7.7<br>5.7	0.89<br>0.87	Robust
	d. Column Temperature (±5 °C)	30 °C	25 °C<br>35 °C	7.7<br>7.7	0.91<br>0.91	Robust
3	a. Flow rate (±0.1 mL·min^{-1})	1.0 (mL·min^{-1})	0.9 (mL·min^{-1})<br>1.1 (mL·min^{-1})	11.0<br>8.9	0.93<br>0.91	Robust
	b. Detection wavelength (±5 nm)	210 (nm)	205 (nm)<br>215 (nm)	9.9<br>9.9	0.94<br>0.91	Robust
	c. Mobile Phase composition: Acetonitrile contents (±5%)	60:40 *v*/*v*	55:45 *v*/*v*<br>65:35 *v*/*v*	13.8<br>7.3	0.91<br>0.89	Robust
	d. Column Temperature (±5 °C)	30 °C	25 °C<br>35 °C	10.4<br>10.4	0.93<br>0.93	Robust

Table S19. *Cont.*

Compound	Parameters	Optimized	Used	Retention Time (min)	Peak Asymmetry	Remarks
4	a. Flow rate (± 0.1 $mL \cdot min^{-1}$)	1.0 ($mL \cdot min^{-1}$)	0.9 ($mL \cdot min^{-1}$)	16.8	1.00	Robust
			1.1 ($mL \cdot min^{-1}$)	13.6	1.09	
	b. Detection wavelength (±5 nm)	210 (nm)	205 (nm)	15.1	0.91	Robust
			215 (nm)	15.1	1.10	Robust
	c. Mobile Phase composition: Acetonitrile contents (±5%)	60:40 *v*/*v*	55:45 *v*/*v*	23.0	1.01	Robust
			65:35 *v*/*v*	10.3	1.01	
	d. Column Temperature (±5 °C)	30 °C	25 °C	15.5	1.00	Robust
			35 °C	15.5	1.01	
5	a. Flow rate (±0.1 $mL \cdot min^{-1}$)	1.0 ($mL \cdot min^{-1}$)	0.9 ($mL \cdot min^{-1}$)	17.6	1.10	Robust
			1.1 ($mL \cdot min^{-1}$)	14.3	1.10	
	b. Detection wavelength (± 5 nm)	210 (nm)	205 (nm)	15.9	1.10	Robust
			215 (nm)	15.9	1.10	
	c. Mobile Phase composition: Acetonitrile contents (±5%)	60:40 *v*/*v*	55:45 *v*/*v*	24.1	1.10	Robust
			65:35 *v*/*v*	10.9	1.01	
	d. Column Temperature (±5 °C)	30 °C	25 °C	16.3	1.10	Robust
			35 °C	16.3	1.10	

Table S20. Analytical results of precision and accuracy.

Compound		Intraday Precision				Intermediate Precision				Interday Precision			
	Conc. (μg·mL^{-1})	Peak Area (mAU*S)	%RSD	Retention Time (min)	%RSD	Peak Area (mAU*S)	%RSD	Retention Time (min)	% RSD	Peak area (mAU*S)	%RSD	Retention Time (min)	%RSD
1	12.5	605.80	0.64	5.30	0.00	605.4	0.09	5.29	0.02	605	0.65	5.29	0.08
	25	1257.8	0.21	5.29	0.15	1257.8	0.00	5.29	0.02	1257.8	0.15	5.30	0.18
	50	2513.6	0.06	5.30	0.21	2513.6	0.00	5.30	0	2513.6	0.08	5.30	0.16
	100	4999.2	0.04	5.30	0.16	4998.7	0.01	5.30	0.05	4998.2	0.10	5.31	0.18
	200	9561.6	0.02	5.31	0.26	9560.1	0.02	5.30	0.05	9558.6	0.02	5.30	0.25
2	12.5	631.20	0.17	7.31	0.18	630.70	0.11	7.31	0.05	630.2	0.13	7.30	0.14
	25	1227.2	0.06	7.30	0.11	1224.9	0.26	7.30	0.01	1222.6	0.10	7.30	0.07
	50	2531.2	0.03	7.30	0.07	2531.6	0.02	7.30	0.01	2532	0.04	7.30	0.07
	100	5201.2	0.02	7.30	0.00	5201	0.00	7.30	0.00	5200.8	0.01	7.30	0.00
	200	10970.2	0.00	7.31	0.20	10970.1	0.00	7.32	0.07	10970	0.01	7.32	0.07
3	12.5	625	0.00	9.99	0.05	624.8	0.04	10.00	0.14	624.6	0.08	10.01	0.47
	25	1214.2	0.09	10.0	0.04	1214.1	0.01	10.00	0.00	1214	0.05	10.00	0.04
	50	2527.2	0.06	10.0	0.44	2527	0.01	10.02	0.00	2526.8	0.05	10.02	0.44
	100	5197.6	0.01	10.0	0.00	5197.7	0.00	10.01	0.14	5197.8	0.00	10.02	0.44
	200	10967.8	0.01	10.0	0.46	10967.7	0.00	10.01	0.01	10967.6	0.00	10.01	0.47
4	12.5	16.0	0.00	15.11	0.07	16.0	0.00	15.11	0.01	16.0	0.00	15.11	0.08
	25	31.0	2.28	15.09	0.34	30.6	1.84	15.09	0.00	30.2	1.48	15.09	0.34
	50	55.8	0.80	15.12	0.27	55.7	0.25	15.11	0.09	55.6	0.98	15.10	0.05
	100	115.4	0.77	15.10	0.05	115.5	0.12	15.10	0.00	115.6	0.47	15.10	0.05
	200	241.8	0.18	15.09	0.34	241.9	0.05	15.08	0.00	242.0	0.00	15.08	0.33
5	12.5	57.78	1.44	16.00	0.03	57.95	0.41	16.00	0.01	58.12	1.84	16.0	0.02
	25	111.82	0.42	16.00	0.00	111.9	0.10	16.00	0.03	111.98	0.63	16.0	0.11
	50	225.40	0.48	16.00	0.10	225.22	0.11	16.00	0.03	225.04	0.53	16.0	0.03
	100	504.54	0.09	16.00	0.03	504.31	0.06	16.00	0.01	504.08	0.12	16.0	0.08
	200	1030.84	0.08	16.00	0.08	1031.12	0.03	16.01	0.00	1031.4	0.13	16.0	0.13
